# Impacts of suicide bereavement on men: a systematic review

**DOI:** 10.3389/fpubh.2024.1372974

**Published:** 2024-04-09

**Authors:** Nina Logan, Karolina Krysinska, Karl Andriessen

**Affiliations:** Centre for Mental Health and Community Wellbeing, Melbourne School of Population and Global Health, The University of Melbourne, Melbourne, VIC, Australia

**Keywords:** suicide bereavement, grief, males, mental health, psychosocial outcomes, suicide, gender

## Abstract

**Introduction:**

This systematic review examines the impacts of suicide bereavement on men’s psychosocial outcomes relating to suicidality, mental health, substance use, grief, and social functioning. Given the high global incidence of suicide and the substantial number of individuals affected by each suicide, understanding the specific experiences and outcomes for men is crucial, particularly in the context of observed gender differences in suicide rates, grief coping styles and mental health outcomes.

**Methods:**

Adhering to PRISMA guidelines, this review included peer-reviewed, English-language studies that involved men bereaved by suicide using quantitative, qualitative and mixed-methods designs. Searches were conducted in MEDLINE, Embase, Emcare, PsycINFO, and Scopus. Analysis used narrative synthesis methods due to the heterogeneity of findings. These were categorised based on comparison groups: non-bereaved men, or women bereaved by suicide. Prospero registration: CRD42023437034.

**Results:**

The review included 35 studies (25 quantitative, 8 qualitative, 2 mixed-methods) published between 1995 and 2023. Compared to non-bereaved men, suicide-bereaved men are more likely to experience adverse psychosocial outcomes included increased suicide mortality, heightened susceptibility to mental health problems such as depression and posttraumatic stress disorder, and challenges in interpersonal relationships and social functioning. The review also identified gender differences in grief responses and coping strategies, with men often exhibiting more pronounced grief reactions and facing unique challenges due to societal expectations and norms regarding masculinity.

**Discussion:**

The findings of this review underscore the elevated risk of adverse suicide- and mental-health related outcomes for suicide-bereaved men and the need for tailored postvention supports for this cohort. Gender-specific factors, including cultural norms and coping strategies, significantly influence men’s experiences of suicide bereavement. Further qualitative and longitudinal quantitative exploration is needed to enhance understanding and effective support for men bereaved by suicide.

**Systematic Review Registration:**

https://www.crd.york.ac.uk/prospero/display_record.php?ID=CRD42023437034.

## 1 Introduction

Suicide bereavement, sometimes referred to as suicide survivorship, entails experiences of grief, distress, and adjustment felt by partners, family, friends, and others close to an individual who has died by suicide. While highly heterogenous, meta-analysis suggests that about one of every 20 people are exposed to suicide each year, with lifetime prevalence of suicide bereavement affecting one of every five people (1). Complementing estimates suggest that, on average, each suicide death affects between 6 family members and 135 people in the community (2). Given the World Health Organization’s recent estimation of approximately 703,000 suicides annually (3), it can be inferred that close to ten million individuals are impacted by the suicide of someone they know each year.

The impacts of a suicide death ripple outwards, affecting individuals to varying degrees based on their proximity and relationship to the deceased (4, 5). Literature often differentiates between the experiences of those closely connected to the deceased, termed *suicide bereavement,* and those less closely connected but still affected, called *suicide exposure* (6). Cerel and colleagues proposed a continuum of survivorship from exposed; to affected; to suicide-bereaved in the short-term; to suicide-bereaved in the long-term (5). Mitchell and colleagues expand on this by noting that the closer an individual is to the deceased, the more significant the impact of a death by suicide on their mental health and quality of life tends to be (7). The familial relationship to the deceased significantly predicts the severity of grief reactions following suicide bereavement, with the most adverse outcomes being strongly linked to the loss of a first-degree relative, including a child, spouse, sibling, or parent. (4). However, unrelated individuals who had close relationships to the deceased can also face adverse impacts similar to the bereaved family (8–10).

There are parallels between the impact of suicide bereavement and bereavement by other means. Common grief reactions include rumination about the deceased person and feelings of sadness, loneliness, emptiness, and anger. Many bereaved individuals describe their experience using the metaphor of “the hole,” indicating the profound absence of the deceased in their lives (11). Others refer to bereavement as “the journey,” highlighting the evolving and varying trajectories of grief. The Dual Process Model of bereavement, developed by Stroebe and Schut, describes this dynamic nature of grief, illustrating how bereaved individuals navigate between loss-oriented stressors—the direct emotional confrontation and process of grief—restoration-oriented stressors, which pertain to the secondary adjustments made in one’s life following bereavement (12).

However, those bereaved by suicide may experience more pronounced grief reactions than other bereaved individuals. They often grapple with heightened guilt or a sense of responsibility for not preventing the suicide (13). The perception that the deceased chose to take their own life can heighten the illusory culpability experienced by those bereaved and leave them contemplating the reasons behind the suicide (13). Bereavement by suicide can increase one’s susceptibility to suicidal behaviour and mortality, especially in immediate family members and partners (4) as well as close friends to the deceased (14). Furthermore, the risk of suicidal behaviours for suicide-bereaved individuals may be greater than for other bereaved individuals (4).

Suicide bereavement is also associated with a range of adverse mental health outcomes including a greater risk of depression, posttraumatic stress disorder (PTSD), and utilisation of psychiatric care (4, 15). Individuals bereaved by suicide often encounter major challenges in their interpersonal relationships. They may face stigmatising attitudes from their community including blame for the suicide, negative reactions ranging from morbid fascination to outright social rejection, or a withdrawal of social support (16, 17). This can prompt some individuals to retreat socially to avoid conversations about the death (16). The impacts of suicide bereavement also extend into professional and educational environments. Pitman and colleagues described the diminished concentration and motivation experienced by bereaved individuals, exacerbating existing bereavement-related stress when employers or educators lack understanding about these effects (18). They also described varied responses to such grief, with some individuals seeking compassionate leave or breaks from education, while others immersed themselves in work or study as a coping mechanism. In the context of the Dual Process Model (12), both the emotional complexity and stigmatised nature of suicide bereavement may intensify the loss- and restoration-oriented stressors. This may create challenges for those experiencing this type of bereavement to express and process their grief, and to engage with the changes in their lives.

Many of the factors impacted by suicide bereavement including suicidal behaviour, mental health outcomes, the expression of grief and emotional reactions, and shifts in interpersonal dynamics and community participation are highly gendered. The global age-standardised suicide mortality rate for men is over twice that for women (3) and a recent systematic review (19) evidenced the continuing validity of the “gender paradox” of suicidology (20); that women are at higher risk of non-fatal suicidal behaviour while men are at higher risk of suicide death. Grief literature highlights differences in prolonged grief, mechanisms of coping and adjustment, and rates of mental health conditions such as depression and PTSD between bereaved men and women (21–25). These gendered variations may be at least partly attributable to the cultural norms that shape responses to bereavement. Notions of masculinity upheld by many cultures emphasise the management of one’s problems without external support. This can heighten self-stigma and reduce help-seeking among men experiencing mental health problems (26). Such norms can also hinder men from building supportive social networks or engaging with therapeutic models that usually focus on talking through feelings of grief (27). Masculine ideals, especially those of self-reliance, are also associated with higher risks of suicidal ideation (28). Given men’s higher risk of suicide mortality and lower help-seeking for mental health problems, there is concern about the impact of suicide bereavement for this population (27). These impacts likely differ for men and women due to these gender differences in mental health- and suicide-related risk factors and may be further affected by diverse patterns often observed between genders in the use of coping mechanisms and grief processing methods (23, 25). However, to date there has been no systematic review of literature concerning the impacts of suicide bereavement specifically for men.

Systematic reviews that explore the impacts of suicide bereavement rarely mention the differential experiences of men and women (4, 9, 29). Review of sampling in suicide bereavement research found a pronounced gender imbalance due most studies including 60–90% female participants (30). This has led to a notable gap in our understanding of the characteristics of suicide bereavement for men. This review is the first to systematically investigate literature regarding the impacts of suicide bereavement for men on psychosocial aspects such as suicidal behaviour, mental health outcomes, grief experiences, social functioning, and substance use. The review also includes findings on physical health, acknowledging the psychosomatic morbidities of suicide bereavement (31).

## 2 Methods

The review protocol was registered with the International Prospective Register of Systematic Reviews (PROSPERO; CRD42023437034) before initiating full-text screening. The review was conducted adhering to the PRISMA (Preferred Reporting Items for Systematic Reviews and Meta-Analyses) guidelines (32), and the research question was structured using the PICO framework (33), identifying the Population (people bereaved by suicide), Intervention (influencing factors), Comparison (with various bereaved or non-bereaved groups), and Outcomes (grief, mental health, social functioning, suicidal behaviour). This guided our focused search strategy and informed the inclusion and exclusion criteria.

### 2.1 Inclusion and exclusion criteria

Studies were included if: (i) the population comprised people bereaved by suicide; (ii) they utilised quantitative, qualitative, or mixed-methods; (iii) they reported data on the psychosocial characteristics of men bereaved by suicide, including outcomes related to grief, mental health, social functioning, or suicidal behaviour; (iv) they were published in English in a peer-reviewed journal; (v) they compared men bereaved by suicide to non-bereaved men, men bereaved by other means, suicide-bereaved women, or did not use a comparator.

Studies were excluded if: (i) the population did not include people bereaved by suicide; (ii) they utilised other methods such as systematic review or case study; (iii) they did not report suicide bereavement-related data on men; or (iv) they were not available as full-text, in English, published in a peer-reviewed journal.

Studies rarely differentiate between cisgender men, transgender men, and other individuals categorised as male in large datasets. Therefore, the term “men” in this review encompasses all groups, with an acknowledgment that samples likely consist predominantly of cisgender men.

### 2.2 Search strategy

**Figure 1:**
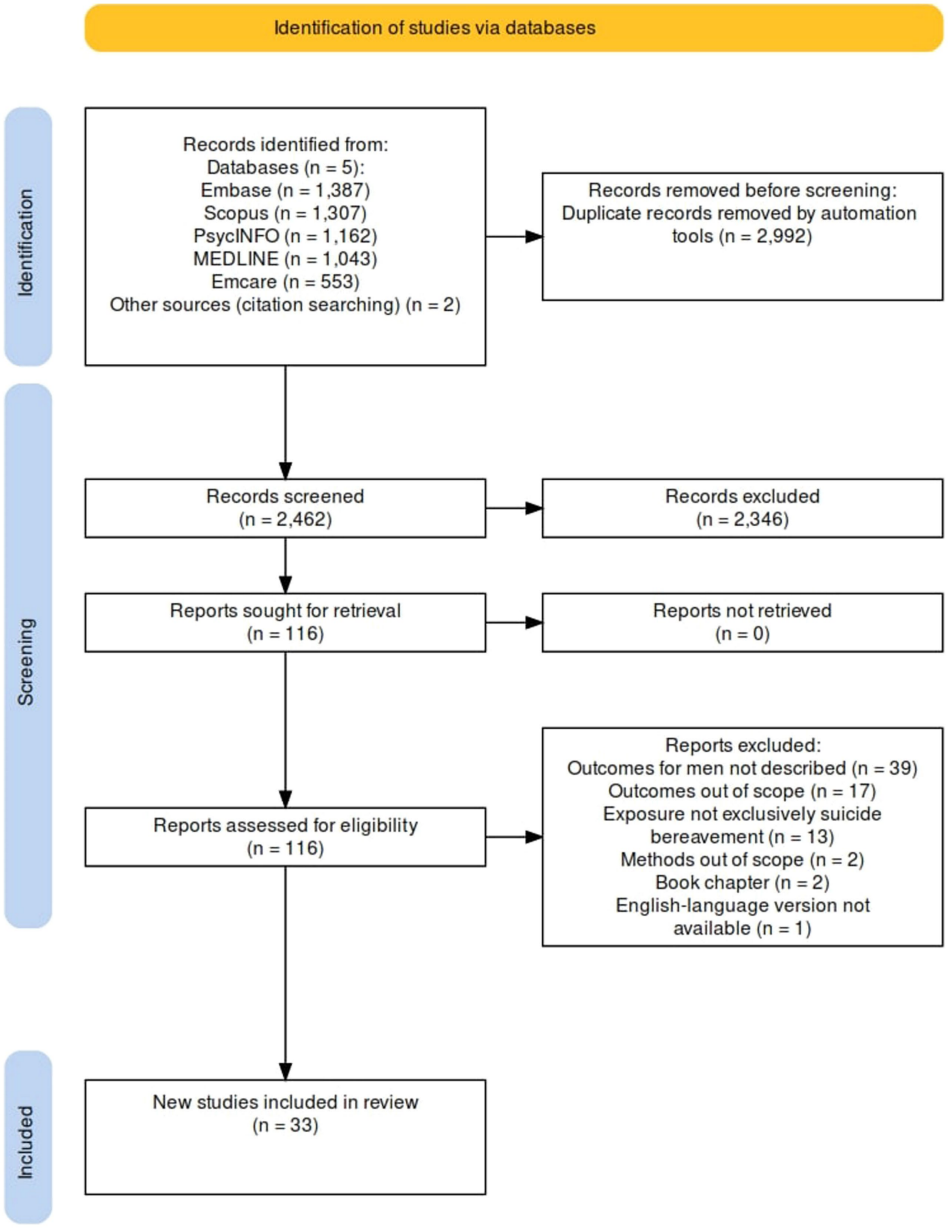
PRISMA diagram of original search.

Researcher NL conducted searches on 21 April 2023 in MEDLINE, Embase, Emcare, PsycINFO and Scopus. The PRISMA diagram for this search is depicted in Figure 1. The search strings comprised concepts of suicide, bereavement, and men. MEDLINE was searched with the following combination of MeSH and keywords: (suicid*.mp OR Suicide/) AND (Grief/ OR grie*.mp OR Bereavement/ OR bereave*.mp OR suicide bereave*.mp OR loss by suicide.mp OR bereave* by suicide.mp OR suicide expos*.mp OR suicide loss survivor.mp) AND (Male/ OR male*.mp OR men.mp OR Men/ OR man.mp OR boy*.mp OR Fathers/ OR father*.mp OR Men’s Health/ OR men* health.mp OR Masculinity/ OR masculin*.mp).

**Figure 2:**
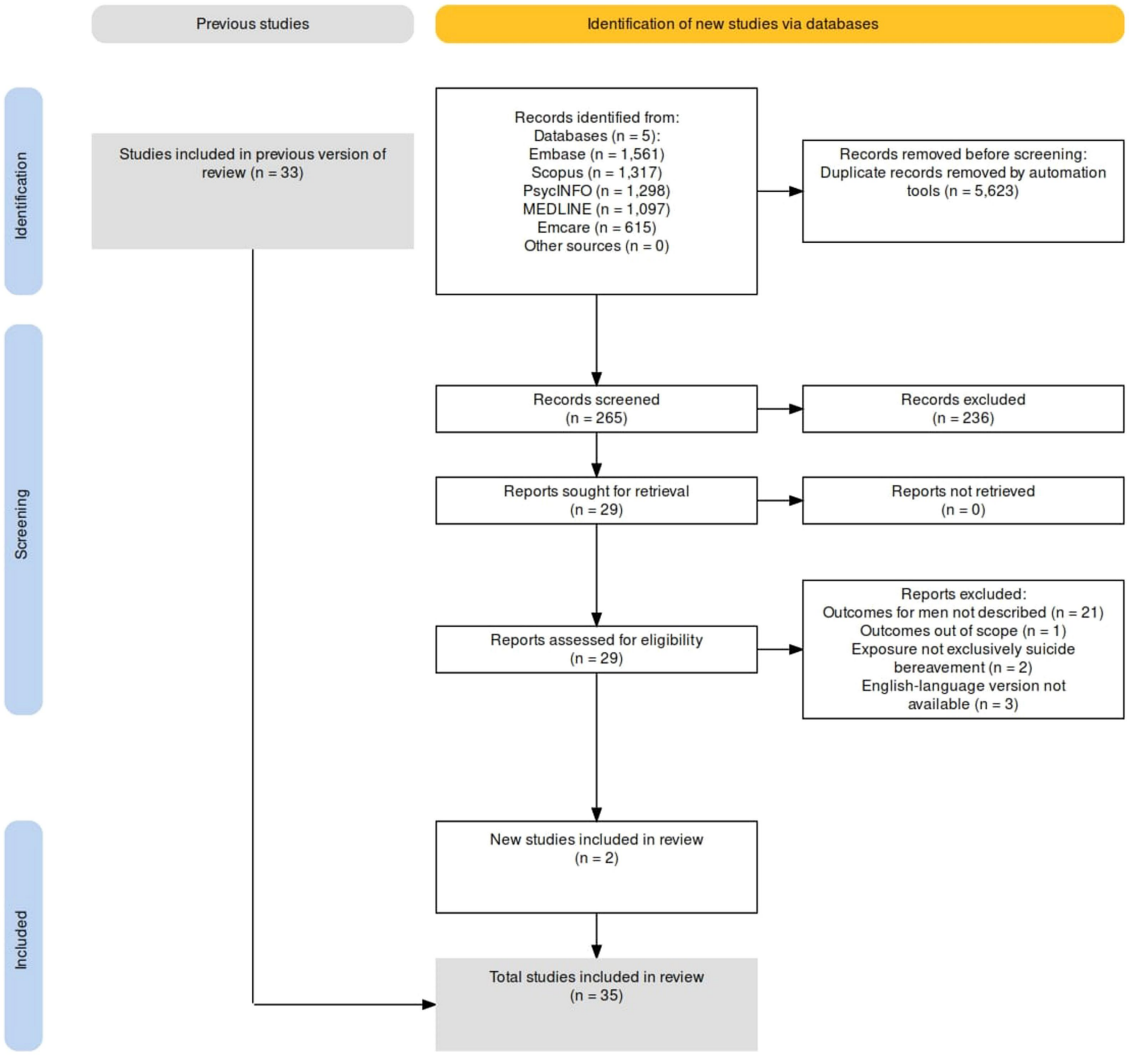
PRISMA diagram of updated search.

The other Ovid-associated databases (Embase, Emcare, PsycINFO) were searched using the same search string, and similar searches were conducted in Scopus. Searches were limited to English-language studies published in peer-reviewed journals, but not by location or date of publication. Eligible articles were subjected to a forward citation search and their references lists were searched to identify additional articles. The database searches were re-run on 16 November 2023, with two additional terms, “suicide survivor*.mp” and “bereave* by *al suicide.mp” included. The PRISMA diagram for the updated search is depicted in Figure 2.

### 2.3 Study selection

Researcher NL imported all records into Covidence, a collaborative, web-based systematic review programme (34), and removed duplicate records. Reviewer NL screened the titles and abstracts of the studies to assess their potential eligibility. Researchers NL and KA independently screened full-text articles of potentially relevant studies to confirm eligibility. Disagreement regarding the inclusion of three articles was resolved through consultation with a third researcher, KK. Figures 1, 2 present the search and selection process.

### 2.4 Data extraction

The research team created a data extraction form including the following variables: study design, eligibility criteria, sample size, age of participants, time since bereavement, relationship to the deceased, comparator or control group, outcome and outcome measure, and main findings. Researchers NL and KK independently extracted data from the included studies, and they resolved any discrepancy through discussion.

### 2.5 Quality assessment of included studies

Evaluation of the quality and potential bias in included studies was conducted using the Mixed Methods Appraisal Tool (MMAT) (35). This tool provides distinct criteria for different types of studies: qualitative studies, quantitative randomised controlled trials, quantitative non-randomised studies, and quantitative descriptive studies. For mixed-methods studies, the MMAT requires evaluation based on the criteria for each specific method used within the study and additional criteria exclusive to mixed-methods designs. Overall scores were summarised for each method based on the suggestions of Hong with a star rating based on the number of quality criteria met (36). Researchers NL and KA independently assessed each study, using MMAT criteria to evaluate the clarity of research questions, appropriateness of methodology and study design, sampling and data collection methods, and the integrity of interpretations made based on the data. Any disagreement was resolved through discussion and consultation with researcher KK.

### 2.6 Data synthesis

Due to the heterogeneity of the included studies, meta-analysis was not suitable. We adopted narrative synthesis instead, as this method allowed for descriptive analysis of results from qualitative, quantitative, and mixed-method studies, using textual explanations. We adhered to the Enhancing Transparency in Reporting the Synthesis of Qualitative Research (ENTREQ) statement for qualitative synthesis and the Synthesis Without Meta-analysis (SWiM) guidelines for quantitative synthesis (37, 38). We synthesised qualitative and quantitative findings separately. Meta-analysis of effect estimates was not possible due to limited reporting of significance, incompleteness of the estimates and utilisation of different effect measures across studies. In alignment with recommended methods of statistical synthesis without meta-analysis, quantitative findings regarding the same outcomes were synthesised using vote counting based on the direction of effect. Findings were included regardless of reported significance or absence thereof (39). Included full-text qualitative studies were imported into NVivo R1 (40) for thematic synthesis. Adopting an iterative process involving researchers KK and KA, researcher NL coded the themes, supporting quotations, and conclusions in these texts. Research NL then examined the coded information to identify patterns related to men’s experiences of suicide bereavement. These were inductively extracted and organised hierarchically into overarching themes (41).

## 3 Results

### 3.1 Study characteristics

The searches yielded 35 studies on 31 samples across various countries (Table 1), including 25 quantitative, eight qualitative and two mixed-methods studies published between 1995 and 2023. The data extracted from the quantitative, qualitative and mixed-methods studies are included in Supplementary Tables 1–4.

**Table 1:** Included studies by region and country.

Region (n)	Country (n)	Citation
Europe (14)	UK (4)	(42–45)
	Denmark (3)	(46–48)
	Sweden (3)	(49–51)
	Germany (1)	(52)
	Ireland (1)	(53)
	Italy (1)	(54)
	Norway (1)	(55)
	Portugal (1)	(56)
North America (13)	Canada (2)	(57, 58)
	United States (11)	(59–69)
Asia (4)	Korea (3)	(70–72)
	Hong Kong (1)	(73)
Oceania (3)	Australia (3)	(74–76)

### 3.2 Samples

Sample sizes of the included quantitative studies (*n* = 25) varied, with individuals bereaved by suicide ranged from 60 (59, 60) to 15,607 (46). Two studies sampled only suicide decedents, with sizes ranging from 9011 (47) to 29,513 (48). Of the studies reporting gender distribution, most (*n* = 17) had fewer than half male participants (42, 47, 49, 50, 52, 54, 55, 59–66, 70, 71) with only seven having gender parity or being predominantly male (46, 48, 51, 56, 67–69). Participant relationships to the deceased varied (Table 2). Mean age ranged from 20 (67) to 54 years (46), with two studies (67, 68) including those under 18. Mean time since bereavement spanned from 1 month (59) to 15 years (77).

**Table 2:** Participant relationships to deceased in quantitative studies.

Relationship to deceased (n)	Citation
Any relative (13)	(42, 52, 54, 56, 59, 60, 62, 66, 68–71, 77)
Any non-relative (13)	(42, 52, 54, 59, 60, 62, 63, 66–69, 71, 77)
Spouse or domestic partner (7)	(46–48, 54, 60, 62, 69)
Any first-degree relative (4)	(48, 63, 64, 72)
Parent (3)	(49, 50, 65)
Offspring (1)	(55)
Sibling (1)	(51)

Sample sizes for suicide-bereaved individuals participating in included qualitative studies (*n* = 8) ranged from 2 (57) to 346 (43). While one study had a notably lower proportion of men (43), most had a gender-balanced sample (44, 45, 74, 75), and three included only men (57, 58, 73). Studies typically examined a single relationship type such as parents of the deceased (45, 74, 75) and domestic partners (57), with one covering both parents and spouses (73) and another including friends, domestic partners, or family members (58). Two studies omitted participants’ relationships to the deceased (43, 44). Of those that reported on age, most focused on individuals aged 40 years and older (45, 57, 74, 75), with others including those at least 30 (73) and two encompassing ages as young as 18 and 20 years (43, 58).

Two mixed-methods studies were included in this review. Time since bereavement ranged between 15 and 38 months in one study (53) and was not reported in the other study (76). Both studies (53, 76) had a sample size of N = 18, with a slightly lower proportion of men compared to women. One sample comprised friends of the deceased (76), while the other included partners, parents, siblings and offspring (53).

### 3.3 Designs

All included quantitative studies (*n* = 25) were non-experimental with half (*n* = 13) employing a cross-sectional design that either exclusively surveyed individuals bereaved by suicide (42, 52, 54, 59, 60, 62, 64, 77) or included individuals not bereaved by suicide (49, 63, 66, 69, 71). Eight quantitative studies used a cohort study design (46, 50, 51, 55, 65, 68, 70, 72), with an additional two studies using a nested case–control design (47, 48). Two quantitative studies used a case–control design (56, 67).

All included qualitative studies (*n =* 8) undertook thematic analysis of qualitative data. Four studies collected this data using semi-structured interviews (45, 73–75), two with photovoice techniques (57, 58), and one as part of a realist evaluation (44). An additional study analysed free-text survey responses (43).

Of the two included mixed-methods studies, one used a concurrent design that collected qualitative and quantitative data in the same timeframe (76). The other used an embedded sequential design with qualitative data being collected from a subsample of participants from a large cohort study (53). Both studies undertook thematic analysis of the qualitative data and descriptive analysis of the quantitative data (53, 76).

### 3.4 Outcomes

Outcomes addressed by the included quantitative studies encompassed six topics related to suicide bereavement: suicidality of the bereaved (42, 46–48, 51, 54, 56, 64, 66, 68, 69, 71, 72), mental health and substance use (46, 49, 52, 54, 56, 59, 60, 65–67, 69, 70, 77), grief (59, 60, 62, 64, 66, 77), relationships and quality of life (42, 54, 56, 60, 63, 64, 69), physical health (46, 59, 60, 70), and employment (46, 50). These topics are documented further in Table 3.

**Table 3:** Outcomes investigated by quantitative studies.

Topic	Outcome (n)	Citation
Suicidality of the bereaved	Suicide ideation and behaviours (8)	(42, 46, 54, 56, 64, 66, 69, 71)
	Death by suicide (5)	(47, 48, 51, 68, 72)
Mental health and substance use	General wellbeing or psychological distress (6)	(46, 52, 54, 56, 59, 60)
	PTSD (6)	(46, 59, 65–67, 77)
	Depression (6)	(46, 49, 56, 60, 66, 77)
	Anxiety (4)	(46, 56, 66, 77)
	Self-harm or non-suicidal self-injury (2)	(46, 69)
	Psychiatric hospitalisation (2)	(46, 70)
	Substance use (2)	(42, 46)
	Use of psychological therapies (1)	(46)
Grief	Complicated or prolonged grief (6)	(59, 60, 62, 64, 66, 77)
	Coping styles (1)	(60)
Relationships and quality of life	Quality of life (2)	(54, 60)
	Adverse life events (2)	(42, 56)
	Social relationships (2)	(64, 69)
	Religion (1)	(63)
	Risky behaviour (1)	(42)
Physical health	General physical health (3)	(46, 59, 60)
	Hospitalisation for physical health condition (2)	(46, 70)
	All-cause mortality (1)	(46)
	Receipt of disability pension (1)	(46)
	Diabetes (1)	(70)
	Cardiovascular disease (1)	(70)
	Cirrhosis (1)	(46)
	Spinal disc herniation (1)	(46)
Employment	Extended sick leave (2)	(46, 50)
	Unemployment (2)	(46, 55)

While all included qualitative studies explored the experiences of suicide bereavement, some focused on specific aspects such as grief (57, 73, 74), coping mechanisms (43, 45, 57, 74, 75), relationships with others (45, 57, 58, 74), emotional repression and masculinities (44, 58, 73), and substance use (43, 74, 75).

Of the included mixed-methods studies, one examined general health, substance use, coping styles, prolonged grief and posttraumatic growth (76). Both studies examined severity of depression and anxiety (53, 76). Qualitative outcomes for one study explored how participants coped with bereavement (76), while the other focused on the impacts of grief, support needs and wellbeing (53).

### 3.5 Quality assessment

The quality and risk of bias assessment of the included studies is summarised in Table 4. The details of the assessment are presented in Supplementary Tables 5, 6. Most qualitative studies (*n* = 17) were of high quality, achieving a star rating of 4–5 (46–51, 55, 56, 62, 63, 66, 68–72, 77). Those that achieved a star rating of 2–3 largely scored poorly on domains relating to representativeness and accounting for confounders in design and analysis (52, 54, 59, 60, 67). The qualitative studies were also of high quality with 63% achieving a 5-star rating (44, 45, 58, 74, 75). All studies with less than five stars did not fulfil the criterion relating to adequacy of data collection methods (43, 57, 73). This was largely due to the potential for selection bias being unaddressed. Regarding the mixed-methods studies, their qualitative elements of both were of high quality (53, 76). There was risk of selection bias in the quantitative elements of one study (76) and both did not adequately investigate divergences in the qualitative and quantitative components (53, 76).

**Table 4:** Summary of quality and risk of bias appraisal of quantitative studies.

Citation	Author/s, Year	Design	Star rating*
Quantitative studies			
(47)	Agerbo, 2005	Nested case–control	★★★★★
(55)	Bélanger et al., 2022	Cohort	★★★★★
(62)	Callahan, 2000	Cross-sectional	★★★★★
(70)	Cho et al., 2016	Cohort	★★★★★
(46)	Erlangsen et al., 2017	Cohort	★★★★★
(63)	Feigelman et al., 2019	Cross-sectional	★★★★★
(72)	Jang et al., 2022	Cohort	★★★★★
(49)	Omerov et al., 2013	Cross-sectional	★★★★★
(48)	Pitman et al., 2022	Nested case–control	★★★★★
(51)	Rostila et al., 2014	Cohort	★★★★★
(50)	Wilcox et al., 2015	Cohort	★★★★★
(77)	Cerel et al., 2017	Cross-sectional	★★★★
(69)	Hom et al., 2017	Cross-sectional	★★★★
(68)	Feigelman et al., 2016	Cohort	★★★★
(71)	Lee et al., 2013	Cross-sectional	★★★★
(56)	Santos et al., 2015	Case–control	★★★★
(66)	van de Venne et al., 2020	Cross-sectional	★★★★
(67)	Brent et al., 1995	Case–control	★★★
(54)	Entilli et al., 2021	Cross-sectional	★★★
(64)	Feigelman et al., 2023	Cross-sectional	★★★
(59)	Mitchell & Terhorst, 2017	Cross-sectional	★★★
(65)	Murphy et al., 1999	Cohort	★★★
(52)	Schneider et al., 2011	Cross-sectional	★★
(60)	Terhorst & Mitchell, 2011	Cross-sectional	★★
(42)	McDonnell et al., 2022	Cross-sectional	0 stars
Qualitative studies			
(44)	Adshead & Runacres, 2022	Realist evaluation	★★★★★
(74)	Entilli et al., 2021	Semi-structured interviews	★★★★★
(45)	Gibson et al., 2010	Semi-structured interviews	★★★★★
(58)	Oliffe et al., 2018	Photovoice	★★★★★
(75)	Ross et al., 2018	Semi-structured interviews	★★★★★
(57)	Ferlatte et al., 2019	Photovoice	★★★★
(73)	Chan & Cheung, 2022	Semi-structured interviews	★★★
(43)	Eng et al., 2019	Free-text survey responses	★★★
Mixed-methods studies			
(53)	Spillane et al., 2018	Embedded mixed-methods (thematic analysis of interviews; case–control study)	★★★★
(76)	Bartik et al., 2020	Concurrent mixed-methods (thematic analysis of interviews; descriptive qualitative study)	★★★

*Quality ranges from 0 to 5 based on the star rating derived from the MMAT (36) with 0 stars being lowest quality and 5 stars being highest.

### 3.6 Findings

#### 3.6.1 Quantitative studies

##### 3.6.1.1 Comparison of suicide-bereaved men to non-suicide-bereaved men

This section documents the direction of effect and statistical significance, where available, of the investigated outcomes for men impacted by suicide bereavement. It compares measures of association for these outcomes to those of men not bereaved by suicide. The quantitative synthesis is presented as an effect direction plot in Table 5. The details of these effect estimates are presented in Supplementary Table 7.

**Table 5:** Effect direction plot summarising direction of impact of suicide bereavement on psychosocial outcomes for men.

Category	Outcome	Relationship to deceased	Erlangsen et al., 2017	Jang et al., 2022	Cho et al., 2016	Rostila et al., 2014	Agerbo et al., 2005	Pitman et al., 2022
Mortality	Suicide mortality	Spouse or partner	△ _**L**_	▲_M_			△ _M_	
		Parent		▲ _M_			◁▷ _M_	
		Sibling				△ _**L**_		
		Any first-degree relative						△ _M_
	All-cause mortality	Spouse or partner	△ _**L**_					
		Sibling				△ _**L**_		
Mental health and substance use	Any mental health problem	Spouse or partner	△ _**L**_					
	Mood disorders	Spouse or partner	△ _**L**_					
	PTSD	Spouse or partner	△ _**L**_					
	Anxiety disorders	Spouse or partner	△ _**L**_					
	Deliberate self-harm	Spouse or partner	△ _**L**_					
	Alcohol use disorder	Spouse or partner	△ _**L**_					
	Drug use	Spouse or partner	△ _**L**_					
	Psychiatric hospitalisation	Spouse or partner	△ _**L**_					
		Family member			△_S_^a^ ▽_S_^b^			

Effect direction: upward arrow ▲ = positive association, downward arrow ▼ = negative association, sideways arrow = no change/conflicting findings.

Sample size: arrow with L subscript ▲_L_ >1,000,000 (large); arrow with M subscript ▲_M_ 50,000-1,000,000 (medium); arrow with S subcript ▲_S_ >50,000 (small).

Statistical significance: black arrow ▲ = *p* <0.05; grey arrow ▲ = *p* > 0.05; empty arrow △ = significance not reported.

PTSD; posttraumatic stress disorder.

^a^ Initial or recurrent psychiatric hospitalisation among men with no prior psychiatric conditions.

^b^ Initial or recurrent psychiatric hospitalisation among men with prior psychiatric conditions.

###### 3.6.1.1.1 Suicide mortality and behaviours

Five large population registry-based cohort or nested case–control studies indicated a positive association between suicide bereavement and increased suicide mortality in men (46–48, 51, 72), with one indicating a stronger association for men bereaved by suicide than those bereaved by other causes (48). A smaller cohort study of adolescent and young men in the US found that participants that died by suicide had significantly higher rates of a family member’s suicide in the year prior (68). Friends’ prior deaths by suicide were not associated with deaths by suicide of participants in this study. A Korean cross-sectional study (71) reported no significant impact on suicidal behaviours among men bereaved by suicide, regardless of their relationship with the deceased.

###### 3.6.1.1.2 Mental health and substance use

A Danish population registry-based cohort study (46) found that men bereaved by a partner’s suicide had an 80% higher adjusted rate of mental health problems over 5 years following bereavement, compared to non-bereaved men. Specifically, PTSD incidence was 12 times higher, and mood disorders, anxiety disorders and self-harm rates were approximately twice as high. Additionally, suicide-bereaved men were five times more likely to use psychological therapy and twice as likely to be hospitalised for psychiatric reasons. This study also reported a 50% higher incidence of alcohol use disorder and 70% higher rate of drug use disorder for men bereaved by a partner’s suicide (46). A Korean cohort study (70) indicated that men bereaved by a family member’s suicide were twice as likely to be hospitalised for psychiatric issues initially or recurrently compared to non-bereaved men. Men with previous psychiatric conditions had a higher initial hospitalisation risk but a lower recurrent hospitalisation risk.

###### 3.6.1.1.3 Quality of life and relationships

Erlangsen and colleagues observed that Danish men bereaved by a spouse’s suicide were over three times more likely to have their children placed outside the home and to receive municipal family support compared to non-bereaved men (46). A US-based survey found no significant differences in religious beliefs or participation between men bereaved by the suicide of a close relative or friend and those who were not (63).

###### 3.6.1.1.4 Physical health

Two population registry-based studies from Denmark and Sweden reported that men bereaved by a spouse, partner, or sibling’s suicide had approximately 30% higher all-cause mortality rates than non-bereaved men (46, 51). Cho and colleagues (2016) found that while the initial hospitalisation risk for cardiovascular disease was similar for men in South Korea bereaved by a family member’s suicide and non-bereaved men, the risk of recurrent hospitalisation was higher for the bereaved (70). Suicide-bereaved men in this study also faced higher risks of both initial and recurrent hospitalisation for diabetes. However, those with pre-existing conditions had a lower hospitalisation risk compared to their non-bereaved counterparts. Erlangsen and colleagues reported that men in Denmark bereaved by a partner’s suicide experienced higher rates of somatic hospitalisation, disability pension receipt, and health conditions like cancer, sleep disorders, liver cirrhosis, and spinal disc herniation (46). Notably, their rates of diabetes were lower, and rates of cardiovascular disease and chronic lower respiratory tract diseases were similar to non-bereaved men.

###### 3.6.1.1.5 Employment and work

A Norwegian population registry-based study found no difference in odds of unemployment between men bereaved by parental suicide and non-bereaved men (55). Conversely, Erlangsen and colleagues noted that Danish men bereaved by a partner’s suicide had higher rates of unemployment and extended sick leave than non-bereaved men (46). Wilcox and colleagues observed that Swedish fathers bereaved by their child’s suicide were more likely to miss work due to psychiatric conditions compared to non-bereaved fathers, an estimated risk similar to fathers bereaved by accidental deaths but twice as high as those bereaved by natural deaths (50). Additionally, suicide-bereaved fathers were more likely to be absent from work due to physical illness, unlike those bereaved by the natural or accidental deaths of their children.

##### 3.6.1.2 Comparison of suicide-bereaved men and suicide-bereaved women

This section documents the direction of effect and statistical significance, where available, of the investigated outcomes for men impacted by suicide bereavement. It compares measures of association for these outcomes to women bereaved by suicide. The quantitative synthesis is presented as effect direction plots in Table 5.

###### 3.6.1.2.1 Suicide mortality and behaviour

Research that compares the suicide mortality risks of suicide-bereaved men and women present mixed findings. Agerbo found that among suicide-bereaved individuals in Denmark, men face a suicide mortality risk three times higher than that of women (47). However, subsequent studies (46, 48, 51, 72) indicate smaller gender disparities. Specifically, research from Denmark and South Korea found that men experience a smaller increase in suicide mortality risk following the suicide of a spouse or domestic partner’s suicide than women (46, 72). This pattern was similarly observed in Swedish men bereaved by a sibling’s suicide compared to women (51). In contrast, Pitman and colleagues who examined the effects of bereavement from the suicide of any first-degree relative, spouse, or domestic partner, reported a higher increase in suicide mortality risk for men than women (48). Despite these varying results, of the studies that assessed the statistical significance of gender differences in suicide mortality risk, whether substantial (47) or modest (46, 48, 72), none of them found the differences to be statistically significant.

Studies investigating the prevalence of suicide attempts and ideation among suicide-bereaved individuals also found varied results by gender. A survey on US military service members and veterans showed no gender differences in rates of suicidal ideation, planning, attempts, or symptoms (69). Similarly a Portuguese study (56) and a UK survey (42) reported no gender variance in suicidal ideation and attempts among suicide-bereaved individuals. However, an Italian study found a lower incidence of suicidal ideation in suicide-bereaved men, compared to suicide-bereaved women (54). These findings should be contextualised by a range of design issues including the employment of self-selection sampling (42, 54, 56) and lower fractions of male participants (42, 54).

###### 3.6.1.2.2 Grief responses

Studies of suicide-bereaved cohorts tended to find lower levels of grief responses in men than women. In Germany, men reported less emotional distress than women (52). In the US, surveyed men displayed lower prolonged grief scores (66) and men in a crisis support intervention used social support and cognitive emotional regulation less frequently than women (60). Two other US-based surveys found no gender differences in grief levels among suicide-bereaved support group members (62), and did not find gender to be a significant predictor of grief-related problems (64).

###### 3.6.1.2.3 Mental health and substance use

Erlangsen and colleagues compared rate ratios of mental health conditions between men and women. They found higher adjusted incident rate ratios of any psychiatric disorder, mood disorders, PTSD, anxiety disorder, drug use disorder, and psychological therapy in suicide-bereaved men than women (46). They also found lower rate ratios for alcohol use disorder and psychiatric hospitalisation.

Other studies explored gendered comparisons of actual rates of mental health distress and conditions among suicide-bereaved individuals. In Portugal, no significant gender differences were found in distress, depression, anxiety, or hostility (56). US studies revealed men were less likely to experience anxiety (66, 77) and had lower depression scores compared to women (66). Swedish research found lower depression prevalence in fathers than mothers bereaved by a child’s suicide (49). Conversely, Feigelman and colleagues did not find gender to be a significant predictor of depressive symptoms among suicide-bereaved individuals (64). With regard to PTSD, studies in the US found a higher proportion of males not developing PTSD in cohorts of suicide-bereaved adolescents (67) and fewer suicide-bereaved fathers met PTSD criteria than mothers (65). However, van de Venne and colleagues found no significant gender differences in PTSD scores in this cohort (66).

###### 3.6.1.2.4 Quality of life, relationships, and physical health

In Italy, a study found no significant gender differences in impacts on participants’ life satisfaction after bereavement by suicide (54). A UK survey indicated that suicide-bereaved men were more likely to engage in high-risk behaviours than women, but reported fewer family or financial problems (42). Additionally, these men experienced fewer health declines and lower prescription drug use than suicide-bereaved women.

#### 3.6.2 Qualitative findings

##### 3.6.2.1 Grief and suicidality

Men who have lost a spouse or partner to suicide often encounter feelings of intense guilt and self-blame, stemming from a perceived inability to prevent the suicide, and feelings of indirect responsibility for the death (57, 73). One such study described experiences of loneliness, depression and suicidality following the suicide of a partner (57). Despite this emotional distress, men in both studies were reticent to seek help due to intersecting identity-based (57) and cultural (73) stigma. Fathers bereaved by the suicide of a child often continued grappling with the profound impact of their loss and exhibited more polarised responses compared to mothers (74). While some continued to struggle with overwhelming grief, others described finding meaning or peace with the death. Ferlatte et al. highlighted how suicide-bereaved men can experience posttraumatic growth, gaining insight into their own resilience and inner strength (57).

##### 3.6.2.2 Coping mechanisms

Studies that explored the use of alcohol to cope with grief reported diverse findings. Among parents bereaved by the suicide of an offspring, some fathers were still using alcohol excessively 2 years following the death while others had significantly reduced their alcohol consumption (74). Another study found no differences around themes of alcohol or drug use by gender (43). Similar variation was found in the experience of continuing bonds with the deceased. Two studies that interviewed the same sample at one- and two-year intervals following bereavement heard how suicide-bereaved fathers preserved connection with their child by maintaining a journal or composing letters (74, 75). However, a study of gay men bereaved by their partner’s suicide heard how some men needed to detach completely from the deceased (57). Some studies noted that bereaved fathers and partners of the deceased coped with their grief by engaging in excessive work hours (57, 74). In contrast, other studies highlighted instances where fathers experienced a loss of motivation for work or re-evaluated the significance of work in relation to other aspects of their lives (45).

##### 3.6.2.3 Relationships with others

Some men experienced significant isolation following bereavement, either imposed by others or themselves. Ferlatte et al. described how men bereaved by the suicide of their partner felt abandoned by their social networks and were unable to initiate new romantic relationships due to lingering trauma from their partner’s death (57). Both this study and another that included gay men (58) found that participants felt compelled to conceal their grief as their families were not aware of their sexuality or relationship. Several studies reported how men bereaved by the suicide of an offspring or close friend had a strong desire to take care of and support their female partners, describing themselves as the “emotional protector” (58, 74). Some expressed deep concern for their spouse and surviving offspring, and a fear of additional loved ones dying by suicide (45), while other men felt that these loved ones were more significantly impacted by the death than themselves (74).

##### 3.6.2.4 Emotional repression and masculinities

Several studies described how suicide-bereaved men often suppressed their emotional responses to bereavement, adopting a more detached grief reaction due to entrenched societal norms around male emotional expression (44, 58). Male participants in a Canadian study contrasted this expectation with how women typically expressed feelings of grief, emphasising that they experienced the same depth of emotions but struggled to identify and reveal them (58). Men in a Hong Kong study also highlighted their deliberate emotional repression as a method to protect their families from the weight of their mourning (73), a theme echoed by the Canadian men who attributed their emotional repression to masculine ideals of stoicism and the importance of self-control to safeguard their loved ones from their own grief (58). However, some men bereaved by the suicide of another male also reflected on how such gender norms intensified the pressures that deceased males had experienced before their suicide and how the experience had emboldened them to seek help for their own mental health challenges (58).

#### 3.6.3 Mixed methods studies

##### 3.6.3.1 Mental and physical health

Spillane and colleagues examined the impacts of suicide bereavement on individuals’ mental and physical health. They found no significance statistical different in levels of depression, anxiety and stress between men and women, nor did themes from semi-structured interviews around grief, psychological and physical conditions vary by gender (53). Bartik and colleagues reported similar findings with no gender differences identified across the wellbeing and physical health of suicide-bereaved adolescent and young adults (76).

##### 3.6.3.2 Coping mechanisms and grief experiences

Qualitative findings from Bartik and colleagues about experiences of bereavement, coping using drugs and alcohol, and support needs did not vary by gender. Young and adolescent men bereaved by the suicide of a friend were described as more likely to use avoidance- and emotion-oriented coping than the standardised male norms (76). Their scores for emotion-oriented coping, social diversion-related coping, and posttraumatic growth appeared to be lower than the young women in the sample, but the sample was too small for statistical analysis. Young men’s levels of posttraumatic growth were significantly lower than standardised male norms, but similar patterns were observed for the women in the sample (76).

## 4 Discussion

### 4.1 Suicide

Suicide-bereaved men are at greater risk of suicide mortality than non-bereaved men (46–48, 51, 72). There is also emerging evidence that they are more at risk of suicide than men bereaved by other means (48). Current impacts of suicide bereavement on suicide mortality risk appear to be similar between men and women (46, 48, 51, 72). However, suicide-bereaved men still died by suicide at higher rates than suicide-bereaved women, reflecting the overall higher suicide risk for men (78). The findings of this review are consistent with other research that suggests that spousal bereavement by any means, is linked to an increased risk of suicide for men that is comparable to that of women (79). Findings from smaller observational studies on gender differences in suicide ideation among suicide-bereaved individuals were mixed. Some reflected the “gender paradox” (80) of men experiencing lower rates of suicide ideation despite having higher rates of suicide mortality (54, 71), while others described no gender differences in risk of suicide ideation among suicide-bereaved individuals (42, 56, 69). However these studies used small samples from a single community (56) or occupation (69), or that were self-selecting (42, 54), which may not reflect patterns outside of these samples.

The qualitative findings, while also susceptible to selection bias, had very few instances of suicide-bereaved men discussing their own suicidality. This was possibly unexpected given the higher risks of suicidality for suicide-bereaved men reflected in quantitative findings. Some men who had lost other males to suicide described how this experience, and in particular seeing the impacts of the loss on other family and friends of the deceased, emboldened them to seek help for their own mental health challenges (58). The only finding to mention suicidality in bereaved men was Ferlatte et al., who described suicide ideation experienced by gay man whose partner had died by suicide during a bereavement process complicated by stigma related to his sexuality and his partner’s HIV positive status. This reflects other findings on the impacts of social marginalisation on bereavement (81) as well as the heightened risk of suicidality for the LGBTIQ+ community (82).

### 4.2 Grief

The quantitative studies included in this review suggest that suicide-bereaved men exhibit lower levels of emotional distress and prolonged grief compared to women, but low response rates may limit these findings’ broader applicability (52, 66). Furthermore, their interpretation must consider the timing of data collection. Examining experiences of prolonged grief among spoused bereaved by a death by any means, Lundorff and colleagues indicated that similar proportion of men and women experience prolonged grief, but with markedly different grief trajectories (22). Men generally experienced a greater intensity of prolonged grief symptoms immediately following bereavement while women showed an increase over time. Thus, the results of this review could be influenced by the predominance of studies that gathered data from participants sometime after bereavement, thereby omitting the initial post-loss phase, a period when men’s grief difficulties might have been most pronounced.

Gender differences in coping strategies for suicide bereavement identified by this review, such as men’s lower utilisation of emotional regulation strategies (60, 76) reflect patterns described by other literature (23). However, such literature also highlights how men may not recognise or report their coping behaviours as forms of emotional regulation. Indeed, qualitative findings describe how suicide-bereaved men regulate or even suppress their emotional responses, influenced by cultural norms of grief expression (73), or a desire to shield others from their own mourning (58). Such avoidance or seeking of distraction from grief emotions may be seen as a maladaptive reaction at first, but can also be part of the natural oscillations of more adaptive coping processes depicted by Stroebe and Schut’s Dual Process Model of coping with bereavement (12). Longitudinal studies with suicide-bereaved men are needed to examine their coping styles and grief outcomes with consideration to the way that these experiences may shift over time.

While studies often describe alcohol use as a common coping strategy for bereaved men (25), findings from this review were more mixed. Some studies reported that suicide-bereaved men utilised typical masculine coping strategies involving alcohol, drugs and gambling more commonly than suicide-bereaved women (42), while others reported that suicide-bereaved women had higher rate ratios of alcohol-related disorders than men (46). Qualitative studies noted that some men used alcohol to cope with the death of a child by suicide (74) but did not expressly note that this was different to women and may be a reflection of the authors’ awareness of men’s alcohol use to cope being a common theme in bereavement-related literature. Other studies found no differences in alcohol use by gender (43, 53, 76).

Further research is needed to examine other aspects of suicide-bereaved men’s grief experiences such as continuing bonds (57, 74, 75) and posttraumatic growth (57, 76) as this review did not yield sufficient and consistent findings to draw any clear conclusions.

### 4.3 Mental health

While only a single study compared mental health outcomes for suicide-bereaved and non-suicide-bereaved men, it found that men bereaved by spousal suicide were at a greater risk of common mental health conditions such as depression, anxiety, and PTSD than non-suicide-bereaved men (46). They were also at greater risk of drug and alcohol use disorders and were more likely to use mental health therapies and services, including psychiatric hospitalisation (46, 70). Studies that only compared risks of depression, anxiety and PTSD in suicide-bereaved men and women largely found the men were at comparatively lower risk of these outcomes (49, 65, 66, 77, 83). This may be driven by gender differences in the risks of these conditions in the broader population where women generally have higher rates of depression, anxiety and PTSD than men (84–86).

Conversely, gender comparisons of rate ratios in Erlangsen et al. found that the rate ratio for PTSD was higher in suicide-bereaved men compared to non-bereaved men, indicating a stronger association between suicide bereavement and PTSD in men than in women, who showed a lower rate ratio when comparing bereaved to non-bereaved individuals. This disparity may also be attributed to typically lower baseline rates of mental health conditions among men, suggesting that the impact of suicide bereavement is more pronounced against a backdrop of lower overall mental health issues among men. However research on spousal bereavement, not specific to suicide, also suggests that bereaved men experience poorer mental health outcomes compared to bereaved women (87).

### 4.4 Quality of life and relationships

Across a range of quality of life and social outcomes, including life satisfaction and family functioning, suicide bereavement appeared to have a negative effect on these aspects of men’s lives to a lesser extent than they did women’s (42, 46, 74). In other outcomes, such as religious participation, suicide bereavement did not appear to have a differential effect by gender (63). Loneliness is often experienced by those grieving a suicide loss (88), and for men, particularly those at greater risk of social isolation such as gay men, it can be more pronounced (57, 58). Some suicide-bereaved men can find purpose in their social networks by focusing on their role as protector of their family and supporting others in their grief (54, 58). However, others used this coping style to avoid addressing their own grief and emotional expression. While these men did not describe this suppression as harmful, it aligns with typical masculine experiences of subduing the emotions of grief both to avoid the stigma of inappropriate emotions and to comfort others (89).

### 4.5 Physical health

Men bereaved by the suicide of a spouse or partner, or sibling, were at greater risk of death by any cause than non-bereaved men (46, 51). Men bereaved by suicide also may be at higher risk of hospitalisation for physical health conditions, however these findings were mixed based on how often they were hospitalised and the conditions for which they were hospitalised (46, 70). Findings were also mixed regarding the effect of gender on the association between suicide bereavement and a range of physical health conditions. While population-level studies observed similar patterns for suicide-bereaved men and women compared to non-suicide bereaved men and women (46), other studies found men to experience fewer adverse physical health effects following suicide bereavement (42). Research on bereavement by any means suggests that this experience can be associated with adverse changes to physical health (90) and the findings of this review generally reflect this.

### 4.6 Strengths and limitations

This review is the first to explore the impacts of suicide bereavement on men. It includes a broad range of outcomes, including suicide mortality and suicidal behaviours, mental health conditions and service utilisation, relationships with others and quality of life, experiences in the workplace, and physical health. The review systematically investigates and synthesises results specific to men across suicide bereavement literature. While studies have used a variety of measures and methods, this review investigates and reports both their agreement and heterogeneity while acknowledging the extent to which its conclusions can be considered reliable. It provides a comparative analysis by contrasting findings from suicide-bereaved men with non-bereaved men and suicide-bereaved women. The included qualitative findings offer in-depth understanding of the emotional and psychological landscapes of suicide-bereaved men, as well as the ways that these aspects may differ from statistical representations. The review also takes an intersectional approach, exploring the different ways that culture and sexuality can impact men’s experiences of suicide bereavement. This review adheres to SWiM guidelines for reporting and discussing its findings (38). It describes and justifies its grouping of studies by comparator, uses quantitative synthesis methods appropriate to heterogenous effect estimates (such as vote counting), investigates heterogeneity of reported effects, uses appropriate data presentation methods and reports the limitations of the synthesis methods and included studies.

The review was limited to peer-reviewed studies, identified by a systematic search of the literature. Nonetheless, it is possible that some relevant studies may not have been captured. The review only included findings related to men bereaved by the suicide of someone close to them, and did not include other relationships, such as clinicians bereaved by a patient’s suicide, or that of first responders. Most studies were based in Europe and other Western countries, with only a few studies from other countries. As such, the findings may not be generalisable to men in non-Western countries, though some are specific to men from East Asian nations. Similarly, while this review suggests that some aspects of suicide bereavement to be specific for gay men, the experiences of other men from the LGBTIQA+ community were not elucidated. Identification of the effects of bereavement by suicide compared to bereavement by other means is limited as most studies compared the experiences of suicide-bereaved individuals to non-bereaved individuals. Similarly, findings regarding differences in the experiences of suicide bereavement for men compared to women may be confounded by baseline gender differences in these outcomes. While this study includes some population-level findings that include all suicide-bereaved men in a certain country and time period, many of the smaller studies had markedly lower proportions of male participants and may be subject to selection bias that often arises from the self-selection of study participants (91, 92). Finally, as meta-analysis was not feasible in this review, qualitative synthesis relied on vote counting methods to summarise the direction of effects and associations, which may oversimplify findings and fail to account for the magnitude or statistical significance of the reported outcomes.

## 5 Conclusion

This review offers crucial insights into the complexity of the impacts of suicide bereavement for men, including the factors that may lead to differences in outcomes within this group. The role of these factors, including relationship to the deceased and other aspects of identity including cultural group and sexuality, should be further explored. Furthermore, while some qualitative findings offer valuable perspectives, there is still an absence of men’s voices in suicide bereavement literature. A greater exploration of men’s experiences of suicide bereavement may enhance our understanding of their grief trajectories, coping, help-seeking, and the risk and protective factors for the adverse outcomes identified by this review.

Findings from this review emphasise the breadth of such adverse outcomes, most saliently the risk of suicide mortality. It is vital that men bereaved by the suicide of a close person, including non-kinship relations, can access tailored postvention supports. While it is important that these supports consider coping mechanisms and norms around masculinity that affect men’s experiences of grief, some men may find that these expectations have adverse impacts on their expression and processing of grief. Conversely, some men may draw strength from these traditional roles and their feeling of responsibility to protect and support their loved ones. It is hoped that the findings of this review may inspire practitioners and others developing postvention programmes to acknowledge men’s suicide bereavement experiences, facilitate sharing, foster a sense of belonging, and promote positive mental health outcomes.

## Data availability statement

The original contributions presented in the study are included in the article/Supplementary material, further inquiries can be directed to the corresponding author.

## Author contributions

NL: Conceptualization, Data curation, Formal analysis, Investigation, Methodology, Project administration, Writing – original draft, Writing – review & editing. KK: Conceptualization, Formal analysis, Methodology, Supervision, Validation, Writing – review & editing. KA: Conceptualization, Formal analysis, Methodology, Project administration, Supervision, Validation, Writing – review & editing.

## References

[1] Andriessen K Rahman B Draper B Dudley M Mitchell PB. Prevalence of exposure to suicide: a meta-analysis of population-based studies. J Psychiatr Res. (2017) 88:113–20. doi: 10.1016/j.jpsychires.2017.01.017, PMID: 28199930

[2] Cerel J Brown MM Maple M Singleton M van de Venne J Moore M . How many people are exposed to suicide? Not six. Suicide Life Threat Behav. (2019) 49:529–34. doi: 10.1111/sltb.12450, PMID: 29512876

[3] World Health Organization. Suicide in 2019: Global health estimates. Geneva: WHO (2021).

[4] Pitman A Osborn D King M Erlangsen A. Effects of suicide bereavement on mental health and suicide risk. Lancet Psychiatry. (2014) 1:86–94. doi: 10.1016/S2215-0366(14)70224-X 26360405

[5] Cerel J McIntosh JL Neimeyer RA Maple M Marshall D. The continuum of “survivorship”: definitional issues in the aftermath of suicide. Suicide Life Threat Behav. (2014) 44:591–600. doi: 10.1111/sltb.12093, PMID: 24702241

[6] Andriessen K Krysinska K. Essential Questions on Suicide Bereavement and Postvention. Int J Environ Res Public Health. (2012) 9:24–32. doi: 10.3390/ijerph9010024, PMID: 22470275 PMC3315078

[7] Mitchell AM Sakraida TJ Kim Y Bullian L Chiappetta L. Depression, anxiety and quality of life in suicide survivors: a comparison of close and distant relationships. Arch Psychiatr Nurs. (2009) 23:2–10. doi: 10.1016/j.apnu.2008.02.007, PMID: 19216982

[8] Kolves K Zhao Q Ross V Hawgood J Spence SH de Leo D. Suicide and other sudden death bereavement of immediate family members: an analysis of grief reactions six-months after death. J Affect Disord. (2019) 243:96–102. doi: 10.1016/j.jad.2018.09.018, PMID: 30241027

[9] Andriessen K Draper B Dudley M Mitchell PB. Pre- and postloss features of adolescent suicide bereavement: a systematic review. Death Stud. (2016) 40:229–46. doi: 10.1080/07481187.2015.1128497, PMID: 26678059

[10] Cerel J Maple M Aldrich R van de Venne J. Exposure to suicide and identification as survivor. Results from a random-digit dial survey. Crisis. (2013) 34:413–9. doi: 10.1027/0227-5910/a000220, PMID: 23871953

[11] Rosenblatt P Bowman T. Alternative approaches to conceptualizing grief: a conversation. Bereave Care. (2013) 32:82–5. doi: 10.1080/02682621.2013.812826

[12] Stroebe M Schut H. The dual process model of coping with bereavement: a decade later. OMEGA - J Death Dying. (2010) 61:273–89. doi: 10.2190/OM.61.4.b 21058610

[13] Jordan JR. Lessons learned: forty years of clinical work with suicide loss survivors. Front Psychol. (2020) 11, 11. doi: 10.3389/fpsyg.2020.00766, PMID: 32411052 PMC7201040

[14] Maple M Cerel J Sanford R Pearce T Jordan J. Is exposure to suicide beyond kin associated with risk for suicidal behavior? A systematic review of the evidence. Suicide Life Threat Behav. (2017) 47:461–74. doi: 10.1111/sltb.12308, PMID: 27786372

[15] Tal Young I Iglewicz A Glorioso D Lanouette N Seay K Ilapakurti M . Suicide bereavement and complicated grief. Dialogues Clin Neurosci. (2012) 14:177–86. doi: 10.31887/DCNS.2012.14.2/iyoung, PMID: 22754290 PMC3384446

[16] Azorina V Morant N Nesse H Stevenson F Osborn D King M . The perceived impact of suicide bereavement on specific interpersonal relationships: a qualitative study of survey data. Int J Environ Res Public Health. (2019) 16:1801. doi: 10.3390/ijerph16101801, PMID: 31117207 PMC6572476

[17] Pitman AL Osborn DPJ Rantell K King MB. The stigma perceived by people bereaved by suicide and other sudden deaths: a cross-sectional UK study of 3432 bereaved adults. J Psychosom Res. (2016) 87:22–9. doi: 10.1016/j.jpsychores.2016.05.009, PMID: 27411748 PMC4988532

[18] Pitman A Khrisna Putri A De Souza T Stevenson F King M Osborn D . The impact of suicide bereavement on educational and occupational functioning: a qualitative study of 460 bereaved adults. Int J Environ Res Public Health. (2018) 15. doi: 10.3390/ijerph15040643, PMID: 29614731 PMC5923685

[19] Miranda-Mendizabal A Castellví P Parés-Badell O Alayo I Almenara J Alonso I . Gender differences in suicidal behavior in adolescents and young adults: systematic review and meta-analysis of longitudinal studies. Int J Public Health. (2019) 64:265–83. doi: 10.1007/s00038-018-1196-1, PMID: 30635683 PMC6439147

[20] Canetto SS Sakinofsky I. The gender paradox in suicide. Suicide Life Threat Behav. (1998) 28:1–23. doi: 10.1111/j.1943-278X.1998.tb00622.x 9560163

[21] Alam R Barrera M D’Agostino N DavidB N Schneiderman G. Bereavement experiences of mothers and fathers over time after the death of a child due to Cancer. Death Stud. (2012) 36:1–22. doi: 10.1080/07481187.2011.553312, PMID: 24567992

[22] Lundorff M Bonanno GA Johannsen M O’Connor M. Are there gender differences in prolonged grief trajectories? A registry-sampled cohort study. J Psychiatr Res. (2020) 129:168–75. doi: 10.1016/j.jpsychires.2020.06.030, PMID: 32739617

[23] Nolen-Hoeksema S. Emotion regulation and psychopathology: The role of gender. Annu Rev Clin Psychol. (2012) 8:161–87. doi: 10.1146/annurev-clinpsy-032511-143109 22035243

[24] Pohlkamp L Kreicbergs U Sveen J. Bereaved mothers’ and fathers’ prolonged grief and psychological health 1 to 5 years after loss-a nationwide study. Psychooncology. (2019) 28:1530–6. doi: 10.1002/pon.5112, PMID: 31108000

[25] Stroebe M Stroebe W Schut H. Gender differences in adjustment to bereavement: an empirical and theoretical review. Rev Gen Psychol. (2001) 5:62–83. doi: 10.1037/1089-2680.5.1.62

[26] Seidler ZE Dawes AJ Rice SM Oliffe JL Dhillon HM. The role of masculinity in men’s help-seeking for depression: a systematic review. Clin Psychol Rev. (2016) 49:106–18. doi: 10.1016/j.cpr.2016.09.002, PMID: 27664823

[27] Grad OT Krysinska K Treven M. Suicide Bereavement and Gender In: Andriessen K Krysinska K Grad OT, editors. Postvention in action. Germany: Hogrefe Publishing (2017). 39–49.

[28] King TL Shields M Sojo V Daraganova G Currier D O’Neil A . Expressions of masculinity and associations with suicidal ideation among young males. BMC Psychiatry. (2020) 20:228. doi: 10.1186/s12888-020-2475-y, PMID: 32398056 PMC7218581

[29] Molina N Viola M Rogers M Ouyang D Gang J Derry H . Suicidal ideation in bereavement: a systematic review. Behav Sci. (2019) 9:53. doi: 10.3390/bs9050053, PMID: 31091772 PMC6562884

[30] Maple M Cerel J Jordan J McKay K. Uncovering and identifying the missing voices in suicide bereavement. Suicidol Online. (2014) 5:1–12.

[31] Spillane A Larkin C Corcoran P Matvienko-Sikar K Riordan F Arensman E. Physical and psychosomatic health outcomes in people bereaved by suicide compared to people bereaved by other modes of death: a systematic review. BMC Public Health. (2017) 17:939. doi: 10.1186/s12889-017-4930-3, PMID: 29228916 PMC5725957

[32] Page MJ McKenzie JE Bossuyt PM Boutron I Hoffmann TC Mulrow CD . The PRISMA statement: an updated guideline for reporting systematic reviews. BMJ. (2020) 2021:n71. doi: 10.1136/bmj.n71 PMC8005924 33782057

[33] Richardson WS Wilson MC Nishikawa J Hayward RS. The well-built clinical question: a key to evidence-based decisions. ACP J Club. (1995) 123:A12–3. doi: 10.7326/ACPJC-1995-123-3-A12, PMID: 7582737

[34] Veritas Health Innovation. Covidence systematic review software. (2023) Melbourne; Available from: www.covidence.org

[35] Hong QN Fàbregues S Bartlett G Boardman F Cargo M Dagenais P . The mixed methods appraisal tool (MMAT) version 2018 for information professionals and researchers. Educ Inf. (2018) 34:285–91. doi: 10.3233/EFI-180221

[36] Hong QN. Reporting the results of the MMAT. (2022). (Mixed Methods Appraisal Tool). Available from: http://mixedmethodsappraisaltoolpublic.pbworks.com/w/file/140056890/Reporting%20the%20results%20of%20the%20MMAT.pdf

[37] Tong A Flemming K McInnes E Oliver S Craig J. Enhancing transparency in reporting the synthesis of qualitative research: ENTREQ. BMC Med Res Methodol. (2012) 12:181. doi: 10.1186/1471-2288-12-181, PMID: 23185978 PMC3552766

[38] Campbell M McKenzie JE Sowden A Katikireddi SV Brennan SE Ellis S . Synthesis without meta-analysis (SWiM) in systematic reviews: reporting guideline. BMJ. (2020) 368:l6890. doi: 10.1136/bmj.l6890, PMID: 31948937 PMC7190266

[39] Higgins JPT Thomas J Chandler J Cumpston M Li T Page MJ . Chapter 12: synthesizing and presenting findings using other methods In: Cochrane handbook for systematic reviews of interventions. 1st ed. US: Wiley (2019)

[40] Lumivero. NVivo R1. (2023). Available at: www.lumivero.com

[41] Thomas J Harden A. Methods for the thematic synthesis of qualitative research in systematic reviews. BMC Med Res Methodol. (2008) 8:45. doi: 10.1186/1471-2288-8-45, PMID: 18616818 PMC2478656

[*42] McDonnell S Flynn S Shaw J Smith S McGale B Hunt I. Suicide bereavement in the UK: Descriptive findings from a national survey. Suicide Life Threat Behav. (2022). 52:887–97. doi: 10.1111/sltb.12874 35611626 PMC9790485

[*43] Eng J Drabwell L Stevenson F King M Osborn D Pitman A. Use of alcohol and Unprescribed drugs after suicide bereavement: qualitative study. Int J Environ Res Public Health. (2019) 16. doi: 10.3390/ijerph16214093, PMID: 31652934 PMC6862291

[*44] Adshead C Runacres J. Sharing is caring: a realist evaluation of a social support group for individuals who have been bereaved by suicide. OMEGA - J Death Dying. (2022). doi: 10.1177/00302228211070152 35098795

[*45] Gibson J Gallagher M Jenkins M. The experiences of parents readjusting to the workplace following the death of a child by suicide. Death Stud. (2010) 34:500–28. doi: 10.1080/07481187.2010.482879, PMID: 24482857

[*46] Erlangsen A Runeson B Bolton JM Wilcox HC Forman JL Krogh J . Association between spousal suicide and mental, physical, and social health outcomes: a longitudinal and Nationwide register-based study. JAMA Psychiatry. (2017) 74:456–64. doi: 10.1001/jamapsychiatry.2017.0226, PMID: 28329305 PMC5470398

[*47] Agerbo E. Midlife suicide risk, partner’s psychiatric illness, spouse and child bereavement by suicide or other modes of death: a gender specific study. J Epidemiol Community Health. (2005) 59:407–12. doi: 10.1136/jech.2004.024950, PMID: 15831691 PMC1733072

[*48] Pitman A McDonald K Logeswaran Y Lewis G Cerel J Erlangsen A. Proportion of suicides in Denmark attributable to bereavement by the suicide of a first-degree relative or partner: nested case-control study. Acta Psychiatr Scand. (2022) 146:529–39. doi: 10.1111/acps.13493, PMID: 35999652 PMC9826113

[*49] Omerov P Steineck G Nyberg T Runeson B Nyberg U. Psychological morbidity among suicide-bereaved and non-bereaved parents: a nationwide population survey. BMJ Open. (2013) 3:e003108. doi: 10.1136/bmjopen-2013-003108, PMID: 23996818 PMC3758979

[*50] Wilcox HC Mittendorfer-Rutz E Kjeldgard L Alexanderson K Runeson B. Functional impairment due to bereavement after the death of adolescent or young adult offspring in a national population study of 1,051,515 parents. Soc Psychiatry Psychiatr Epidemiol. (2015) 50:1249–56. doi: 10.1007/s00127-014-0997-7, PMID: 25552253

[*51] Rostila M Saarela J Kawachi I. “The psychological skeleton in the closet”: mortality after a sibling’s suicide. Soc Psychiatry Psychiatr Epidemiol. (2014) 49:919–27. doi: 10.1007/s00127-013-0780-1, PMID: 24126558

[*52] Schneider B Grebner K Schnabel A Georgi K. Is the emotional response of survivors dependent on the consequences of the suicide and the support received? Crisis. (2011) 32:186–93. doi: 10.1027/0227-5910/a000081, PMID: 21940250

[*53] Spillane A Matvienko-Sikar K Larkin C Corcoran P Arensman E. What are the physical and psychological health effects of suicide bereavement on family members? An observational and interview mixed-methods study in Ireland. BMJ Open. (2018) 8:e019472. doi: 10.1136/bmjopen-2017-019472, PMID: 29331974 PMC5781012

[*54] Entilli L Leo D Aiolli F Polato M Gaggi O Cipolletta S. Social support and help-seeking among suicide bereaved: A study with Italian survivors. Omega. (2023). 87:534–53. doi: 10.1177/00302228211024112 34128417

[*55] Belanger SM Stene-Larsen K Magnus P Reneflot A Christiansen SG Hauge LJ. Employment status and bereavement after parental suicide: a population representative cohort study. BMJ Open. (2022) 12:e064379. doi: 10.1136/bmjopen-2022-064379, PMID: 36167366 PMC9516068

[*56] Santos S Campos RC Tavares S. Suicidal ideation and distress in family members bereaved by suicide in Portugal. Death Stud. (2015) 39:332–41. doi: 10.1080/07481187.2014.946626, PMID: 25551259

[*57] Ferlatte O Oliffe JL Salway T Knight R. Stigma in the bereavement experiences of gay men who have lost a partner to suicide. Cult Health Sex. (2019) 21:1273–89. doi: 10.1080/13691058.2018.1556344, PMID: 30644338

[*58] Oliffe JL Broom A Kelly MT Bottorff JL Creighton GM Ferlatte O. Men on losing a male to suicide: a gender analysis. Qual Health Res. (2018) 28:1383–94. doi: 10.1177/1049732318769600, PMID: 29683063

[*59] Mitchell AM Terhorst L. PTSD symptoms in survivors bereaved by the suicide of a significant other. J Am Psychiatr Nurses Assoc. (2017) 23:61–5. doi: 10.1177/1078390316673716, PMID: 27742751

[*60] Terhorst L Mitchell AM. Ways of coping in survivors of suicide. Issues Ment Health Nurs. (2011) 33:32–8. doi: 10.3109/01612840.2011.618584, PMID: 22224964

[*61] Cerel J Maple M van de Venne J Moore M Flaherty C Brown M. Exposure to suicide in the community: prevalence and correlates in one U.S. State Public Health Rep Wash DC 1974. (2016) 131:100–7. doi: 10.1177/003335491613100116 PMC4716477 26843675

[*62] Callahan J. Predictors and correlates of bereavement in suicide support group participants. Suicide Life Threat Behav. (2000) 30:104–24. doi: 10.1111/j.1943-278X.2000.tb01070.x, PMID: 10888052

[*63] Feigelman W Cerel J McIntosh JL Brent D Gutin N. Suicide bereavement and differences in religiosity: much ado about sex. Crisis J Crisis Interv Suicide Prev. (2019) 40:176–85. doi: 10.1027/0227-5910/a000546, PMID: 30215302

[*64] Feigelman W Cerel J Sheehan L Oexle N. Using multiple regression analyses to uncover patterns of correlates of grief problems, depression and suicidal ideation among suicide bereaved individuals. Omega. (2023) 87:554–71. doi: 10.1177/00302228211024812, PMID: 34148402

[*65] Murphy SA Braun T Tillery L Cain KC Johnson LC Beaton RD. PTSD among bereaved parents following the violent deaths of their 12- to 28-year-old children: a longitudinal prospective analysis. J Trauma Stress. (1999) 12:273–91. doi: 10.1023/A:1024724425597, PMID: 10378166

[*66] van de Venne J Cerel J Moore M Maple M. Sex differences in mental health outcomes of suicide exposure. Arch Suicide Res Off J Int Acad Suicide Res. (2020) 24:158–85. doi: 10.1080/13811118.2019.1612800 31081470

[*67] Brent DA Perper JA Moritz G Liotus L Richardson D Canobbio R . Posttraumatic stress disorder in peers of adolescent suicide victims: predisposing factors and phenomenology. J Am Acad Child Adolesc Psychiatry. (1995) 34:209–15. doi: 10.1097/00004583-199502000-00016, PMID: 7896654

[*68] Feigelman W Joiner T Rosen Z Silva C. Investigating correlates of suicide among male youth: questioning the close affinity between suicide attempts and deaths. Suicide Life Threat Behav. (2016) 46:191–205. doi: 10.1111/sltb.12183, PMID: 26247908 PMC7871898

[*69] Hom MA Stanley IH Gutierrez PM Joiner TEJ. Exploring the association between exposure to suicide and suicide risk among military service members and veterans. J Affect Disord. (2017) 207:327–35. doi: 10.1016/j.jad.2016.09.043, PMID: 27743535

[*70] Cho J Jung SH Kim C Suh M Choi YJ Sohn J . Suicide loss, changes in medical care utilization, and hospitalization for cardiovascular disease and diabetes mellitus. Eur Heart J. (2016) 37:764–70. doi: 10.1093/eurheartj/ehv448, PMID: 26371117

[*71] Lee MA Kim S Shim EJ. Exposure to suicide and suicidality in Korea: differential effects across men and women? Int J Soc Psychiatry. (2013) 59:224–31. doi: 10.1177/0020764012441296, PMID: 22433241

[*72] Jang J Park S Kim Y Kim E Lee SJ . Risks of suicide among family members of suicide victims: a nationwide sample of South Korea. Front. Psychiatry. (2022) 13:834. doi: 10.3389/fpsyt.2022.995834 PMC9614235 36311502

[*73] Chan TMS Cheung M. The “men in grief” phenomenon among suicide bereaved Chinese men in Hong Kong. Death Stud. (2022) 46:1845–52. doi: 10.1080/07481187.2020.1855609, PMID: 33306457

[*74] Entilli L Ross V De Leo D Cipolletta S Kolves K. Experiences of parental suicide-bereavement: a longitudinal qualitative analysis over two years. Int J Environ Res Public Health. (2021) 18. doi: 10.3390/ijerph18020564, PMID: 33440875 PMC7826588

[*75] Ross V Kolves K Kunde L De Leo D. Parents’ experiences of suicide-bereavement: a qualitative study at 6 and 12 months after loss. Int J Environ Res Public Health. (2018) 15. doi: 10.3390/ijerph15040618, PMID: 29597297 PMC5923660

[*76] Bartik WJ Maple M McKay K. Youth suicide bereavement and the continuum of risk. Crisis. (2020) 41:483–9. doi: 10.1027/0227-5910/a000653, PMID: 32036701

[*77] Cerel J Maple M van de Venne J Brown M Moore M Flaherty C. Suicide exposure in the population: perceptions of impact and closeness. Suicide Life Threat Behav. (2017) 47:696–708. doi: 10.1111/sltb.12333, PMID: 28150414

[78] World Health Organisation. Suicide worldwide in 2019: Global health estimates [internet]. Geneva: World Health Organisation (2021).

[79] Ajdacic-Gross V Ring M Gadola E Lauber C Bopp M Gutzwiller F . Suicide after bereavement: an overlooked problem. Psychol Med. (2008) 38:673–6. doi: 10.1017/S0033291708002754, PMID: 18226288

[80] Schrijvers DL Bollen J Sabbe BGC. The gender paradox in suicidal behavior and its impact on the suicidal process. J Affect Disord. (2012) 138:19–26. doi: 10.1016/j.jad.2011.03.050, PMID: 21529962

[81] McNutt B Yakushko O. Disenfranchised grief among lesbian and gay bereaved individuals. J LGBT Issues Couns. (2013) 7:87–116. doi: 10.1080/15538605.2013.758345

[82] de Lange J Baams L van Bergen DD Bos HMW Bosker RJ. Minority stress and suicidal ideation and suicide attempts among LGBT adolescents and Young adults: a Meta-analysis. LGBT Health. (2022) 9:222–37. doi: 10.1089/lgbt.2021.0106, PMID: 35319281

[83] Brent DA Moritz G Bridge J Perper J Canobbio R. The impact of adolescent suicide on siblings and parents: a longitudinal follow-up. Suicide Life Threat Behav. (1996) 26:253–9. doi: 10.1111/j.1943-278X.1996.tb00610.x, PMID: 8897664

[84] Christiansen DM Hansen M. Accounting for sex differences in PTSD: a multi-variable mediation model. Eur J Psychotraumatol. (2015) 6:26068. doi: 10.3402/ejpt.v6.26068, PMID: 25604705 PMC4300366

[85] Harkness KL Alavi N Monroe SM Slavich GM Gotlib IH Bagby RM. Gender differences in life events prior to onset of major depressive disorder: the moderating effect of age. J Abnorm Psychol. (2010) 119:791–803. doi: 10.1037/a0020629, PMID: 20853920 PMC3638862

[86] McLean CP Asnaani A Litz BT Hofmann SG. Gender differences in anxiety disorders: prevalence, course of illness, comorbidity and burden of illness. J Psychiatr Res. (2011) 45:1027–35. doi: 10.1016/j.jpsychires.2011.03.006, PMID: 21439576 PMC3135672

[87] Yoon H Park GR Kim J. Psychosocial trajectories before and after spousal loss: does gender matter? Soc Sci Med. (2022) 294:114701. doi: 10.1016/j.socscimed.2022.114701, PMID: 35007946

[88] Pitman AL King MB Marston L Osborn DPJ. The association of loneliness after sudden bereavement with risk of suicide attempt: a nationwide survey of bereaved adults. Soc Psychiatry Psychiatr Epidemiol. (2020) 55:1081–92. doi: 10.1007/s00127-020-01921-w, PMID: 32683472 PMC7395013

[89] Kiaos T. Stop being a wuss: People’s perceptions of men experiencing grief in Australia. Health Promot J Austr. (2023). doi: 10.1002/hpja.794, PMID: 37705129

[90] Carlsson N Alvariza A Bremer A Axelsson L Årestedt K. Symptoms of prolonged grief and self-reported health among bereaved family members of persons who died from sudden cardiac arrest. OMEGA - J Death Dying. (2023) 87:66–86. doi: 10.1177/00302228211018115, PMID: 34011206 PMC10064453

[91] Choi I Milne DN Glozier N Peters D Harvey SB Calvo RA. Using different Facebook advertisements to recruit men for an online mental health study: engagement and selection bias. Internet Interv. (2017) 8:27–34. doi: 10.1016/j.invent.2017.02.002, PMID: 30135825 PMC6096306

[92] Ellis LA McCabe KL Rahilly KA Nicholas MA Davenport TA Burns JM . Encouraging young men’s participation in mental health research and treatment: perspectives in our technological age. Clin Investig. (2014) 4:881–8. doi: 10.4155/cli.14.61

