# Supplementary material for: Impacts of suicide bereavement on men: a systematic review
Source: Front Public Health. 2024 Apr 9;12:1372974. doi: 10.3389/fpubh.2024.1372974 (PMC11035897; doi:10.3389/fpubh.2024.1372974)
Supplement: Supplementary file 1 [file Data_Sheet_1.PDF]

Supplementary Material

Impacts of suicide bereavement on men: A systematic review

Nina Logan*, Karolina Krysinska, Karl Andriessen

*** Correspondence:** Corresponding Author: nina.logan@unimelb.edu.au

1 Supplementary Figures and Tables

1.1 Supplementary Tables

Author (Year), Location	Study design	Eligibility criteria	Sample size (sample size of men bereaved by suicide); Gender ratio (M%:F%)	Age (years) = Range (M, SD) *(T = total, M = male, F = female)*	Time since bereave ment	Relationship to deceased	Comparator	Outcome; outcome measure	Main findings
Agerbo (2005), Denmark	Nested case-control study	Individuals living in Denmark aged 25-60 who died by suicide during 1982-97	Cases = 9011 (24); controls = 180220; M:F = 40:60	AgeT = 25-60	Not reported	Domestic partner or parent	Age- and gender matched individuals who did not die by suicide	Death by suicide	Of those bereaved by the suicide of a partner, men died by suicide (AIRR 46.20; 95CI 18.34-116.40) at a rate 3.05 times higher than women (AIRR 15.75; 95CI 6.64-37.38). This comparison of rate ratios was not significant. Rate ratios of suicide for mothers (AIRR 2.30; 95CI 0.92-5.73) and fathers (AIRR 2.06; 95CI 0.84-5.07) bereaved by the suicide of a child compared to those non-bereaved were similar. Significance of level of the rate ratios was not reported. Adjustment was for age, psychiatric admission,

									number of children, labour market affiliation, and education.
Bélanger (2022), Norway	Observational cohort study using population-based register	All Norwegian residents aged 25-49 between 2000-14	Cases = 3826 (1886); controls = 2264837; M:F = 49:51	AgeT = 25-49 (37.4, 8.3)	Not reported	Offspring	Individuals not bereaved by the suicide of a parent	Employment status	Adjusted odds of non-employment for men bereaved by the suicide of a parent non-bereaved men did not differ significantly (AOR 1.00; 95CI 0.88-1.13; p > 0.05). Adjusted odds of non-employment for women bereaved by the suicide of a parent were significantly higher compared to non-bereaved women (AOR 1.20; 95CI 1.09-1.33; p < 0.01). Adjustment was for age, education, marital status, offspring, and parental education.
Brent (1995), USA	Case-control study	Adolescent friends and acquaintances of adolescents who died by suicide in western Pennsylvania	146 (79); M:F = 54:46	AgeCases = 20.1 (1.5); AgeControls = 18.2 (2.0)	M = 7.3 months, SD = 1.7 months	Friend or acquaintance	Suicide-bereaved individuals that did not develop PTSD	Post-traumatic stress disorder (PTSD); PTSDRI.	0% of those bereaved by suicide who developed PTSD were male, compared to 57.2% of those who did not develop PTSD (p = 0.002).
Callahan (2000), USA	Cross-sectional descriptive study	Participants in bereavement support groups who were bereaved by suicide	210 (41); M:F = 19:81	AgeT = (44.95, 12.86)	M = 4.05 years, SD = 6.65 years	Family member, spouse or other	Not applicable	Grief; GEQ.	No statistical or clinical difference in level of grief experienced by men (135.07) and women (137.04) bereaved by suicide (t = -0.20, p = 0.844).
Cerel (2017), USA [a]	Cross-sectional descriptive study	Individuals who reported suicide exposure in a random-digit dial telephone	807 (365); M:F = 46:54	AgeT = 19-94 (53.55, 14.76)	M = 15.49 years (SD = 13.72 years)	First degree relative, second degree relative, or nonrelative	Individuals not exposed to suicide	Anxiety and depression; PHQ. PTSD; Short Screening Scale for PTSD.	Males exposed to suicide had a lower likelihood of anxiety than females exposed by suicide (OR 0.51; p = 0.01). Exposed males also had a lower likelihood of reporting intermediate and high levels of impact, with exposed females being more likely to experience these higher levels (F(2, 31), p < 0.001).

		survey conducted in Kentucky, USA from 2012-13						Prolonged grief; PG-13.	
Cho (2016), Korea	Observational cohort study	Cases were the family members aged > 40 years of individuals who died of suicide in 2002-2003.	Cases = 4253 (1705); controls = 9467; M:F = 38:62	AgeT > 40	Not reported	Family member	Family members of a control subject age- and gender-matched to the individual who died suicide	Inpatient and outpatient medical care visits for cardiovascular disease (CVD), diabetes (DM), and psychiatric conditions during the year following bereavement by suicide.	After adjusting for age, region, and socioeconomic status, of those without a past history of the outcomes, men bereaved by suicide were at lower risk of initial hospitalisation for CVD (AHR 0.967; 95CI 0.675-1.387) compared to non-bereaved men. However, they were at increased risk (AHR 1.343; 95CI 1.001-1.800) of recurrent hospitalisation for CVD. Men bereaved by suicide were at an increased risk of initial (AHR 3.383; 95CI 1.715-6.673) and recurrent (AHR 2.238; 95CI 1.379-3.632) hospitalisation for DM compared to non-bereaved men. Men bereaved by suicide were also at increased risk of initial (AHR 2.665; 95CI 1.495-4.750) and recurrent (AHR 2.135; 95CI 1.425-3.198) hospitalisation for psychiatric conditions compared to non-bereaved men. However, of those with a past history of the outcomes, men bereaved by suicide were at lower risk of initial (AHR 0.385; 95CI (0.259-0.573) and recurrent (AHR 0.611; 95CI 0.485-0.770) hospitalisation for any of the outcomes.
Entilli (2021), Italy	Cross-sectional	Adults aged >18 years residing in Italy that were bereaved by suicide.	132 (27); M:F = 48:52	AgeT = (42.3, 14.9)	M = 66.2 months, SD = 70.9 months	Close relative, partner or non-relative	Not applicable	Life satisfaction; SWLS. Wellbeing; WHO-5. Ad-hoc questions on prevalence	Male gender was associated with lower suicide ideation (negligible negative correlation, r = -0.17; p = 0.043). Male gender was also associated (negligible positive correlation) with higher life satisfaction (r = 0.11) and wellbeing (r = 0.02), with none of these associations being significant (p > 0.05).

								and frequency of suicide ideation.	
Erlangsen (2017), Denmark	Cohort study using population-based register	Adults aged >18 years bereaved by the suicide of a spouse.	Cases = 15607 (4814); controls = 6991291; M:F = 50:50	AgeM = (54.0, 14.2), AgeF = (49.6, 15.2)	5 years	Spouse	The general population, and individuals bereaved by spousal death by other means	Registered psychiatric or somatic diagnosis, death, records of family changes (divorce, child removal), extended sick leave, employment, receipt of pension, hospitalisation, GP or psychologist appointment	Bereaved men experienced higher adjusted rates of any mental disorder than non-bereaved men (AIRR 1.8; 95CI 1.6-2.0). Adjusted rates were markedly higher for PTSD (AIRR 12.1; 95CI 6.4-22.7), anxiety disorders (AIRR 2.5; 95CI 1.8-3.4), mood disorders (AIRR 2.2; 95CI 1.8-2.7) and deliberate self-harm (AIRR 2.0; 95CI 1.5-2.7). Risks for disorders related to alcohol (AIRR 1.5; 95CI 1.2-1.8) and drug use (AIRR 1.7; 95CI 1.2-2.5) were also higher. Adjusted rates of liver cirrhosis (AIRR 1.6; 95CI 1.1-2.3) and spinal disc herniation (AIRR 1.5; 95CI 1.2-1.9) were higher for bereaved men. The adjusted rate of all-cause mortality was higher (AIRR 1.3; 95CI 1.2-1.4) and the suicide rate was markedly higher (AIRR 6.4; 95CI 5.3-7.8), however this was lower than the adjusted suicide rate ratio for bereaved women compared to non-bereaved women (AIRR 8.5; 95CI 7.0-10.5). Bereaved men’s adjusted rates of child removal (AIRR 3.6; 95CI 2.9-4.5), municipal family support (AIRR 3.7; 95CI 2.8-5.0), extended sick leave (AIRR 2.3; 95CI 2.1-2.5), unemployment (AIRR 2.1; 95CI 1.9-2.3), and disability pension receipt (AIRR 2.9; 95CI 1.8-4.8) were also higher. They also had higher rates of service usage with markedly higher adjusted rates of psychiatric hospitalisation (AIRR 2.7; 95CI 2.1-3.7) and psychological therapy (AIRR 5.8; 95CI 4.6-7.4). Rates of somatic hospitalisation (AIRR 1.2; 95CI 1.0-1.3) and GP contact (AIRR 1.1; 95CI 1.0-1.1)

									were comparable to that of non-bereaved men. Significance levels for these comparisons were not reported. Adjustment was for calendar period, country of birth age, civil status, income, existing physical conditions, prior psychiatric hospitalisation, and record of self-harm.
Feigelman (2023), USA	Cross-sectional survey	Adults bereaved by suicide, primarily in the US	195 (15); M:F = 8:92	AgeT = 18-77 (50.4, 12.9)	1 month - 35 years M = 107.6 months, SD = 88.8	Immediate family member, including spouse, live-in partner, parent, child, or sibling	Not applicable	Perceived suicide stigma; STOSASS-b, secrecy; SLSS, grief difficulties; BGQ, suicidal thoughts; SIDAS, personal growth; PSSQ, depression; CES-D	Gender was initially found to be a statistically significant correlate for grief problems among suicide-bereaved individuals, however it was found to be a redundant predictor when other correlates were considered. Findings were similar when the model considered the gender as a predictor of depression. Gender was not found to be a predictor of suicide ideation for suicide-bereaved individuals.
Feigelman (2019), USA	Cross-sectional survey	Noninstitutionalised adults (>18 years) in the US.	Cases = 515 (180), controls = 912; M:F = 35:65	Not reported	M = 14 years	Friend or first-degree relative	Individuals not bereaved by suicide	Religious beliefs and participation.	There were no significant differences in religious beliefs and participation between bereaved and nonbereaved men.
Feigelman (2016), USA	Retrospective cohort study	Male high school students	10122 (N/A): 100%	AgeM = 13-25	Not reported	Family member or friend	Individuals who did not die by suicide	Death by suicide	Men that had died by suicide had were bereaved by a family member's suicide within the past year at a significantly higher rate than men that did not die by suicide. Friend's deaths by suicide were not associated with men's deaths by suicide.
Hom (2017), USA	Cross-sectional, survey-	US military service members	Cases = 1004, controls =	AgeT = 18-88 (33.44, 13.38)	Not reported	Spouse, partner, family member, friend, fellow	Individuals not exposed to suicide	Current suicidal symptom severity;	No significant difference between males and females exposed to suicide in terms of suicide ideation, plans or attempts, nor self-reported current suicidal symptom.

	based study	and veterans that know someone who died by suicide.	749; M:F = 82:18			service member or other military contact, or other		DSI-SS. Experiences of suicidal thoughts and behaviours, SBQ-R. Perception of lack of meaningful social connection; INQ-TB. Lifetime history of non-suicidal self-injury; NSSI.	Comparisons made by one-way ANOVAs and chi-square tests but results and significance were not reported. Gender did not moderate the association between closeness to the decedent and impact of their death.
Jang (2022), Korea	Population-based cohort study	Immediate family members of individuals who died by suicide in South Korea between 2008-17	Cases = 423331 families, controls = 420978 families	Not reported	M = 25.4 months	Immediate family (spouse, parent, child or sibling)	Age- and gender-matched immediate family members of individuals who died by non-suicidal means	Death by suicide following the death by suicide of an immediate family member	Men bereaved by the suicide of an immediate family member were at significantly greater risk of suicide than non-suicide-bereaved men. For those bereaved by a child's suicide, suicide risk was lower for fathers (HR 1.817, 95CI 1.464-2.256; p<0.0001) than mothers (HR 2.652, 95CI 2.077-3.386; p<0.0001). For those bereaved by a spouse's suicide, suicide risk was lower for husbands (HR 3.871, 95CI 3.165-4.733; p<0.0001) than wives (HR 5.096, 95CI 3.982-6.522; p<0.0001).
Lee (2012), Korea	Cross-sectional study using survey data	Participants in the 2009 Korean General Social Survey who were	1602 (769); M:F = 48:52	AgeT = (43.519, 15.227), AgeM = (42.786,	Not reported	Someone the subject was close to	Individuals not exposed to the suicide of someone close to them	Suicidality; MINI.	Exposure to the completed suicide of someone close was associated with a significant moderate increase in the suicidality of female respondents (moderate positive correlation, r = 0.242; p < 0.001), but no significant change in male respondents (negligible positive correlation, r = 0.066; p > 0.05).

		exposed to the suicide of an individual that they were close to.		(15.148), AgeF = (44.195, 15.278)					
McDonnell (2022), UK	Cross-sectional survey-based descriptive study	Adult (>18 years) UK residents bereaved or affected by suicide.	7158 (1519); M:F = 21:79	AgeT = 18-84 (43.6, 13.0)	< 1 year (21%), 1 – 5 years (36%), 5 – 10 years (15%), 10 – 20 years (15%), > 20 years (12%)	Friend, family member, family-in-law, ex-partner, colleague, or other	Not applicable	Ad-hoc questions on impact of bereavement by suicide and resulting adverse life events; engagement in high-risk behaviours; suicidal behaviours.	Men reported engaging in high-risk behaviours following the bereavement by suicide more commonly than women (33% cf. 29%; p = 0.006). Males reported experiencing fewer adverse social events such as family problems, and financial troubles compared to women (32% cf. 41%; p < 0.001). They also reported experiencing fewer adverse health events, including mental illness, deterioration in physical health, and usage of prescription drugs that women (42% cf. 50%; p <0.001). Men also reported self-harm less often (5% cf. 8%; p <0.001). Events that men did report more often include gambling (2% cf. <1%; p < 0.001), alcohol use (21% cf. 17%; p = 0.001), and illicit drug misuse (9% cf. 4%; p < 0.001). However, there was no significant different between genders in the rates of suicide ideation (36% cf. 38%; p = 0,303) and suicide attempt (9% cf. 8%; p = 0.456).
Mitchell (2017), USA [b]	Cross sectional survey-based study	Adults bereaved by the death by suicide participating in a bereavement crisis intervention	60 (17); M:F = 18:72	AgeT = (43, 14)	1 month	Family member, friend or co-worker	Not applicable	PTSD symptoms; IES. General physical and mental health; MOS SF-36. Complicat	Males reported fewer PTSD symptoms than female, but this was not statistically significant (t = -1.82, p = 0.074, d = -0.53). However, males significantly experienced fewer intrusion symptoms (t = -2.431, p = 0.018, d = -0.70) and total stress (t = -2.22, p = .030, d = -0.43) than females.

								ed grief; ICG.	
Murphy (1999), USA	Prospective analysis of mixed cohort study	Parents of children who died by suicide, were aged between 12-28 at time of death and were not married	261 (90); M:F = 34:66	AgeT = 32-61 (45, 6)	2-7 months (30-month follow up)	Parent	Parents of children who died by accident or homicide.	PTSD symptoms; TES.	Among parents who lost a child to suicide, 36% of mothers and 5% of fathers met PTSD diagnostic criteria. These groups were not compared using a statistical test.
Omerov (2013), Sweden	Population-based case-control survey	Swedish-born parents bereaved by the death by suicide of a child aged 15-30 between 2004-7 in Sweden	Eligible cases = 915, eligible controls = 508. Cases = 666 (283), controls = 377; M:F = 42:58	*Median (IQR):* AgeCasesM = 58 (53-62), AgeCasesF = 55 (51-59). AgeControlsM = 59 (54-62), AgeControlsF = 54 (50-59).	3-6 years	Parent	Non-bereaved parents matched for age, sex, living area, martial status, and number of children.	Depression; PHQ.	Fathers bereaved by suicide had a higher prevalence of moderate-to-severe depression (10%) than non-bereaved fathers (4%), but a lower prevalence than bereaved mothers (23%). Statistical comparisons were not shown in this article, but it was noted that statistically significant differences between bereaved and non-bereaved parents remained after adjusting for sex and a range of other known risk factors.

Pitman (2022), Denmark	Population-based nested case-control study	All Danish-born residents who had not migrated and died by suicide	Cases = 29513 (213); Controls = 117641 (203); M:F = 63:37	AgeT (median, IQR) = 52, 40-66	Not reported	First-degree relative or partner	Age-matched individuals alive on the day of suicide	Death by suicide	Suicide-bereaved men were over three times more likely to die by suicide than non-suicide bereaved men (AOR 3.26; 95CI 2.62–4.06) after adjusting for marital status, family size, household income level, pre-bereavement history of self-harm, mental and physical health conditions. Similar but attenuated odds were found for suicide-bereaved women compared to non-suicide-bereaved women (AOR 2.50; 95CI 1.96–3.18). Suicide-bereaved men were also more likely to die by suicide than men bereaved by other means (AOR 1.49; 95CI 1.19–1.86). Similar odds were found for suicide-bereaved women, compared to women bereaved by other means (AOR 1.45; 95%CI 1.13–1.86). No significance evidence was found to suggest an interaction with gender, either when contrasting suicide bereavement with non-bereavement (p = 0.107), or when compared to non-suicide bereavement (p = 0.896).
Rostila (2013), Sweden	Population register-based cohort study	Individuals born in Sweden during 1932-1962 who were alive at the end of 1980 bereaved by the suicide of a sibling	13643 cases (6833), 1734426 controls; M:F = 50:50	AgeT = 25-64	Not reported	Sibling	Individuals not bereaved by the suicide of a sibling	Death by suicide	Men bereaved by the suicide of a sibling died by suicide at a higher adjusted rate than men not bereaved by suicide (ARR 2.38; 95CI 1.81-3.14), but at a lower rate than women bereaved by the suicide of a sibling (ARR 3.25; 95CI 2.28-4.65). Significance was not reported. Shifts in all-cause mortality rates over time differed between men (ARR 1.26; 95CI 1.14-1.40) and women bereaved by a sibling's suicide, with men seeing a spike in the second year following bereavement and women seeing spikes in the sixth and tenth years. Adjustment was for age, year, socioeconomic status, marital status, number of children, number of siblings, and region.

Santos (2014), Portugal	Case-control study	Individuals bereaved by the death by suicide of a family member that they lived with in Alentejo Central, Portugal	Cases = 93 (31), controls = 102; M:F = 67:33	AgeCasesT = 17-89 (52.6, 18.9), AgeControlsT = 17-89 (48.6, 15.9)	Not reported	Family member	Individuals from the same community not bereaved by suicide	Suicide ideation; SIQ. General distress, depression, anxiety, hostility; BSI.	Male and female participants did not significantly differ regarding general distress, t(91) = 1.74, ns; depression, t(91) = 1.95, ns; anxiety, t(91) = .85, ns; hostility, t(91) = 1.47, ns; and suicidal ideation, t(91) = .31, ns.
Schneider (2011), Germany	Cross-sectional quantitative analysis of semi-structured psychological autopsies	Relatives and friends of individuals who died by suicide in the Frankfurt/Main area in 1999-2000	163 (60); M:F = 37:63	Not reported	M = 8.5 months, SD = 6.8 months	Relative or friend	Not applicable	Emotions following bereavement	Females bereaved by suicide were at higher odds of “any kind of emotions having disturbed everyday life” (OR 2.84; 95CI 0.84-9.66). Taking the reciprocal of this, males were at lower odds of "any kind of emotions having disturbed everyday life" (OR = 0.35; 95CI 0.10-1.19). Significance not reported.
Terhorst (2012), USA [b]	Secondary analysis of cross-sectional survey data	Adults (>18 years) participants in a crisis debriefing intervention who were bereaved by the suicide of a family member or significant other	60 (17); M:F = 28:72	AgeT = (43.3, 13.7)	< 1 month	Close friend, relative or spouse	Not applicable	Coping style, WoCQ. Depression, BDI. Emotional and physical symptoms; BSI. Complicated grief, ICG. Quality of	Males demonstrated a lower utilisation of Seeking Social Support (t = -2.622, d = -0.77, p = .011) and Positive Reappraisal (t = -2.373, d = -0.76, p = .021) coping styles compared to females.

		within the previous month.						life, MOS SF-36.	
van de Venne (2020), USA [a]	Cross sectional analysis of survey data	Participants in a random-digit dial survey conducted 2012-13 in Kentucky, USA	1703 (374); M:F = 46:54	AgeM = 19-101 (52.87, 16.04); AgeF = 19-99 (55.02, 16.55)	Not reported	First degree relative, second degree relative, or non-relative	Not applicable	Anxiety, depression, suicide ideation; PHQ. PTSD, Short Screening Scale for PTS. Prolonged grief, PG-13.	Of those bereaved, on average males scored significantly lower for depression (4.71 cf. 5.81; t(783) = -2.84, p <= 0.01), anxiety (4.01 cf. 5.60; t(802) = -4.21, p < 0.001) and prolonged grief (14.80 cf. 16.46; t(779) = -3.10, p <= 0.01) . No significant gender differences in mean scores for PTSD (0.91 cf. 1.14; t(787) = -1.85, p = 0.065). However, on average males scored significantly higher for suicide ideation (0.16 cf. 0.09; t(672) = 2.319, p > 0.05)
Wilcox (2014), Sweden	Population-based prospective cohort study	Parents of offspring aged 16-24 in Sweden in 2004 who were not at retirement age or receiving a disability pension	Cases = 537 (261), cohort = 1,051,515; M:F = 49:51	AgeM = (52, 5.9); AgeF = (49, 5.9)	Not reported	Parent	Parents of offspring aged 16-24 who did not die by suicide during the study period	Sickness absence from work for greater than 30 days due to psychiatric or somatic diagnoses	After adjusting for age, education, marital status, urbanicity, country of birth, prior sickness absence, disability pension, and prior diagnoses fathers bereaved by suicide were significantly more likely to be absent from work due to psychiatric diagnoses than non-bereaved fathers (AHR 11.2, 95CI 8.9-14.2; p < 0.05). This risk was comparable to fathers bereaved by accidental death (AHR 11.1, 95CI 9.1-13.6; p < 0.05) and mothers bereaved by suicide (AHR 9.9, 95CI 8.4-11.6; p < 0.05), but higher than fathers bereaved by natural death (AHR 4.0, 95CI 2.9-5.5; p < 0.05). Compared to non-bereaved fathers, there were no significant differences in the risk of sickness absence due to somatic diagnoses between either father bereaved by natural or accidental death, or between non-bereaved mothers and mothers bereaved by suicide. However, compared to non-bereaved fathers, fathers bereaved by suicide were significantly more likely to have somatic sickness absence (AHR 1.4, 95CI 1.0, 1.8; p < 0.05).

[a] Cerel (2017) and van de Venne (2020) are part of the same research project and use the same sample for analysis.
[b] Terhorst (2012) and Mitchell (2017) are part of the same research project and use the same sample for analysis.

Supplementary Table 1. Data extraction of quantitative studies

**Author (Year), Location**	**Study design**	**Eligibility criteria**	**Sample size (sample size of men bereaved by suicide); Gender ratio (M%:F%)**	**Age (years) = Range (M, SD)** ***(T = total, M = male, F = female)***	**Time since bereavement**	**Relationship to deceased**	**Outcome**	**Main findings**
Adshead (2022), UK	Realist evaluation	Over 18 years, attending bereavement support group, English speaking, no neurological/developmental/learning disability	6 (3); M:F = 50:50	Not reported	Not reported	Not reported	Experiences and impact of suicide bereavement on wellbeing	Bereavement was (a) contextualised by experiences of external and self-stigma, with men describing the impacts of traditional ideas of masculinity on their expression of grief and need to conceal negative emotion. (b) Mechanisms of progress through bereavement include reluctance to impose support needs and various motivations to overcome grief. (c) Outcomes included the development of a sense of belonging, healthy habits, and hope for the future.
Chan (2022), Hong Kong	Deductive thematic analysis of semi-structured interviews	Adult (over 18 years) men who have been Hong Kong residents for +10 years who	10 (10); M:F = 100:0	Age = 30-60	<2 years	Parent or spouse	Experiences of grief and help-seeking	Three themes characterise the unique cultural and gendered experiences of grief for Chinese men: (a) the emotional challenges and complexity of hidden grief characterised by self-blame, delay in formal help-seeking, and refusal of help from others, (b) processing of guilt and grief complicate by suicide being a social taboo, fear of judgement from others and avoidance of formal and informal supports, (c) grief-related social stigma leading to repression of

		are service users at NGO that supports men bereaved by suicide. Bereaved by spouse or child death by suicide in past two years.						emotions, and expectation for men to be stoic and strong.
Eng (2019), UK	Thematic analysis of free-text response in survey	Survey respondents aged 18-40 years bereaved by the death by suicide of a close friend or relative since the age of 10.	346 (67); M:F = 19:81	Age = 18-40	Not reported	Not reported	Response to question about impact of suicide bereavement on use of alcohol and unprescribed drugs	Three themes were identified: (a) loss of control or greater restriction around drug and alcohol use following suicide bereavement, (b) drug and alcohol use as a coping mechanism, (c) impact of factors other than suicide bereavement on drug and alcohol use. No variation in themes by gender.
Entilli (2021), Australia [a]	Thematic analysis of semi-structured interviews	Parents bereaved the suicide of a child in Queensland, Australia	14 (7); M:F = 50:50	AgeM = 50-68 (60), AgeF = 50-78 (60)	Not reported	Parent	Experiences of sense-making, coping strategies, and finding meaning and purpose following suicide	Three themes were identified at 6 and 12 months (see Ross, 2018). 24 months post-bereavement, shifts were observed in pre-established theme. (a) Parents had increasingly developed personal explanations for the death, however some fathers still grappled with understanding the event, though less dominantly than earlier periods, and expressed surprise at the extent to which they were still struggling. Some fathers' sense of overwhelm and negative mood had increased with time, with important anniversaries heightening the realisation of not having recovered from the loss. Grief was

							bereavement	compounded by concern for their spouse/partner's coping. (b) New adaptive and maladaptive coping strategies had emerged in this period. Several fathers changed their occupations to less stressful ones after the suicide while others coped through excessive work and alcohol consumption.
Ferlatte (2019), Canada[b]	Thematic analysis of photovoice	Gay men bereaved by the death by suicide of romantic partner	2 (2); M:F = 100:0	Age = 40-49	Not reported	Romantic partner	Experiences of suicide bereavement and stigma (both suicide- and identity-based)	Five themes characterised the narrative of suicide bereavement. (a) Recognising the risk of suicide prior to death, reflecting on this as attempts to prevent the inevitable, (b) feelings of failure and guilt to protect their partner from death and the added complexity of the forced "outing" of their sexuality and/or HIV status to family and loved ones following the death, (c) overwhelming pain and loss from their partner's death, particularly when contrasted to hostile relationships with other biological family, (d) isolation in grief due to limited support from social networks already weakened by sexual minority stigma, (e) reluctance to seek professional help, instead focussing on work and creativity as an outlet for grief. Loneliness and depression can precipitate suicidality in the bereaved individual and impact future romantic relationships.
Gibson (2010), UK	Exploratory qualitative analysis of semi-structured interviews	Parents bereaved by the death by suicide of a child over 18 months prior in Northern Ireland	11 (5); M:F = 45:55	Age = 44-57	>18 months	Parent	Experiences in the workplace following suicide bereavement	Parents' experiences were characterised by three major themes; (a) social and emotional challenges relating to interaction with, and judgement from others in the workplace, (b) impacts of bereavement on cognitive, emotional and physical functioning, e.g., a father's work concentration suffered due to concerns about his wife's coping after suicide bereavement, (c) changed attitudes to work and life with gender differences in how parents adjusted to work following the loss. Compared to mothers, who tended to work harder early on as a coping mechanism, fathers showed reduced motivation, workloads, and ambition.

Oliffe (2018), Canada[b]	Photovoice	Men residing in Canada aged >19 years bereaved by the death of a male friend, partner or family member to suicide	20 (20); M:F = 100:0	Age = 20-63 (39)	Not reported	Close friend, romantic partner or family member	Experience of suicide bereavement	Men who have bereaved by the death by suicide of another man can rationalise the experience or alternately perceive the incident as unexpected. The management of emotions related to suicide can catalyse significant life changes. Moreover, the process of discussing male suicide can highlight conflicts around differing ideals of masculinity.
Ross (2018), Australia[a]	Qualitative analysis of semi-structured interviews, part of larger longitudinal study	Parents in Queensland, Australia bereaved by the death by suicide of a child, whose other parent was not participating in the study	14 (7); M:F = 50:50	AgeM = 50-68 (60), AgeF = 50-78 (60)	6 months (first interview) and 12 months (second interview)	Parent	Experiences of aftermath and bereavement by the suicide of a child	Three themes were identified 6 and 12 months post-bereavement (see Entilli, 2021 for themes at 24 months). (a) Both mothers and fathers struggled to make sense of their loss and find reasons for the suicide at 6 and 12 months. (b) Parents at 6 and 12 months used both adaptive and maladaptive coping strategies, with no clear gender differences in strategies used. However, specific examples showed some variation; fathers, for instance, used more avoidance strategies such as working excessively to distract from their grief. A unique adaptive coping strategy among fathers was writing letters to their deceased child. (c) More prevalent at 12 months was some parents coming to terms with the loss and renewed senses of meaning and purpose, while others continued to struggle with grief.

[a] Ross (2018) and Entilli (2021) are part of the same research project and use the same sample for analysis.
[b] Oliffe (2018) and Ferlatte (2019) are part of the same research project and use the same sample for analysis.

Supplementary Table 2. Data extraction of qualitative studies

**Author (Year), Location**	**Study design**	**Eligibility criteria**	**Sample size (sample size of men bereaved by suicide); Gender ratio (M%:F%**	**Age = Range (M, SD) *(T = total, M = male, F = female)***	**Time since bereavement**	**Relationship to deceased**	**Outcome measure *Outcome, outcome measure***	**Main findings**
Bartik (2020), Australia	Concurrent mixed-method design including thematic analysis of interviews and comparison of psychological measures with standardised norms	Young people and adolescents bereaved by the death by suicide of a friend, continued to reside in rural location of friend's death	18 (7); M:F = 39:61	AgeTotal = 14-23 (17, 3); AgeM = 14-23 (17, 2); AgeF = 15-22 (17, 2)	Not reported	Friend	General health; non-standardised Likert. Alcohol use; non-standardised Likert. Coping; CISS. Depression; BDI-II. Anxiety; STAI. Prolonged grief, PG-13. Posttraumatic growth, PGI.	Males were more likely to use avoidance-oriented coping and emotion-oriented coping than the normative samples of males ($t(6)=1.552$; $p=0.172$ and $t(6) = 1.722$; $p=0.136$ respectively) but this was not significant. Compared to females in the same sample, males had lower mean scores for emotion-oriented coping, and social diversion. Males had higher mean scores for avoidance- and distraction-oriented coping. They had lower mean scores for posttraumatic growth. However, these means were not statistically compared in the study. Males in the study also had significantly lower scores for posttraumatic growth than standardised norms for males ($t(6) = -9.548$, $p < 0.001$). Qualitative findings clustered about three themes that did not vary by gender: (a) distorted communication about the death on social media and in-person, (b) mixed responses to the death in schools, socially, and in community, (c) coping with the death using alcohol, drugs, and other risky behaviours.

Spillane (2017), Ireland	Embedded sequential mixed-methods design including qualitative analysis of semi-structured interviews and quantitative analysis of data from a larger case-control study.	Participants in a larger case-control study who were bereaved by a family member's death by suicide	18 (7); M:F = 39:61	Not reported	15-38 months, M = 27.6 months	Partner/spouse, parent, sibling or child	Physical and psychological health effects of suicide bereavement; semi-structured interviews and DASS-21.	No significant difference found in levels of depression (p=0.47), anxiety (p=0.37), and stress (p=0.87) between men and women bereaved by suicide. Three main themes characterised the impacts of bereavement, (a) links between grief, psychological and physical health, (b) lack of fulfilment of support needs, (c) attempts to reconstruct wellbeing and broader life following bereavement. These did not vary by gender.

Supplementary Table 3. Data extraction of mixed-methods studies

Acronym	Name	Citation
**BDI**	Beck Depression Inventory	Beck et al., 1961
**BDI-II**	Beck Depression Inventory II	Beck et al., 1996
**BSI**	Brief Symptom Inventory	Derogatis & Spencer, 1982; Derogatis, 1993
**CISS**	Coping Inventory for Stressful Situations	Endler & Parker, 1990
**DASS-21**	Depression Anxiety and Stress Scale	Lovibond & Lovibond, 1995
**DSI-SS**	Depression Symptom Inventory – Suicidality Subscale	Metalsky & Joiner, 1997
**GEQ**	Grief Experience Questionnaire	Barret & Scott, 1989
**ICG**	Inventory of Complicated Grief	Prigerson et al., 1995
**IES**	Impact of Events Scale	Horowitz et al., 1979
**INQ-TB**	Interpersonal Needs Questionnaire – Thwarted Belongingness Scale	Gutierrez & Joiner, 2016
**MINI**	Mini International Neuropsychiatric Interview	Agoub, Moussaoui & Kadri, 2006; Yoo et al., 2006
**MOS SF-36**	Medical Outcomes Study - Short Form 36	Stewart et al., 1988; Ware et al., 1979; Ware et al., 1993

No acronym	Short Screening Scale for PTSD	Breslau et al., 1999
**NSSI**	Non-Suicidal Self-Injury Measure	Nock et al., 2007
**PG-13**	Prolonged Grief Scale	Prigerson et al., 2009
**PGI**	Posttraumatic Growth Inventory	Tedeschi et al., 1996
**PHQ**	Patient Health Questionnaire	Spitzer et al., 1999
**PTSDRI**	Posttraumatic Stress Disorder Reaction Index	Pynoos et al., 1987
**SBQ-R**	Suicidal Behaviors Questionnaire - Revised	Osman et al., 2001
**SIQ**	Suicide Ideation Questionnaire	Ferreira & Castela, 1999
**STAI**	State Trait Anxiety Inventory	Spielberger, 1989
**SWLS**	Life Satisfaction Scale	Diener et al., 1985; DiFabio et al., 2020
**TES**	Trauma Experiences Scale	Murphy et al., 1999
**WHO-5**	WHO-5 Wellbeing Index	World Health Organisation, 1998
**WoCQ**	Ways of Coping Questionnaire	Folkman & Lazarus, 1985

Supplementary Table 4. Acronym for outcome measures used in Supplementary Tables 1, 2, and 3

**Author (Year), Location**	**S1**	**S2**	**Q.1**	**Q2**	**Q3**	**Q4**	**Q5**
Quality and risk of bias assessment of included quantitative studies using MMAT criteria for quantitative nonrandomised studies (Category 3)							
Agerbo (2005), Denmark	Yes	Yes	Yes	Yes	Yes	Yes	Yes
Bélanger (2022), Norway	Yes	Yes	Yes	Yes	Yes	Yes	Yes
Brent (1995), USA	Yes	Yes	Can’t tell	Yes	Yes	No	Yes
Callahan (2000), USA	Yes	Yes	Yes	Yes	Yes	Yes	Yes
Cerel (2017), USA	Yes	Yes	No	Yes	Yes	Yes	Yes
Cho (2016), Korea	Yes	Yes	Yes	Yes	Yes	Yes	Yes
Entilli (2021), Italy	Yes	Yes	Can’t tell	Yes	Yes	Can't tell	Yes
Erlangsen (2017), Denmark	Yes	Yes	Yes	Yes	Yes	Yes	Yes
Feigelman (2023), USA	Yes	Yes	No	Yes	Yes	No	Yes
Feigelman (2019), USA	Yes	Yes	Yes	Yes	Yes	Yes	Yes
Feigelman, (2016), USA	Yes	Yes	Yes	Yes	Yes	No	Yes

Hom (2017), USA	Yes	Yes	Can’t tell	Yes	Yes	Yes	Yes
Jang (2022), Korea	Yes	Yes	Yes	Yes	Yes	Yes	Yes
Lee (2012), Korea	Yes	Yes	Yes	Yes	Yes	No	Yes
McDonnell (2022), UK	Yes	Yes	No	Can’t tell	Can't tell	No	Can’t tell
Mitchell (2017), USA	Yes	Yes	No	Yes	Yes	No	Yes
Murphy (1999), USA	Yes	Yes	Yes	Can’t tell	Yes	Can't tell	Yes
Omerov (2013), Sweden	Yes	Yes	Yes	Yes	Yes	Yes	Yes
Pitman (2022), Denmark	Yes	Yes	Yes	Yes	Yes	Yes	Yes
Rostila (2013), Sweden	Yes	Yes	Yes	Yes	Yes	Yes	Yes
Santos (2014), Portugal	Yes	Yes	Can’t tell	Yes	Yes	Yes	Yes
Schneider (2011), Germany	Yes	Yes	Can’t tell	Can’t tell	Yes	No	Yes
Terhorst (2012), USA	Yes	Yes	No	Yes	Can't tell	No	Yes
van de Venne (2020), USA	Yes	Yes	No	Yes	Yes	Yes	Yes
Wilcox (2014), Sweden	Yes	Yes	Yes	Yes	Yes	Yes	Yes
Quality and risk of bias assessment of included qualitative studies using MMAT criteria for qualitative studies (Category 1)							
Adshead (2022), UK	Yes	Yes	Yes	Yes	Yes	Yes	Yes
Chan (2022), Hong Kong	Yes	Yes	Can’t tell	No	Yes	Yes	Yes
Eng (2019), UK	Yes	Yes	Yes	No	No	Yes	Yes
Entilli (2021), Australia	Yes	Yes	Yes	Yes	Yes	Yes	Yes
Ferlatte (2019), Canada	Yes	Yes	Yes	Can’t tell	Yes	Yes	Yes
Gibson (2010), UK	Yes	Yes	Yes	Yes	Yes	Yes	Yes
Oliffe (2018), Canada	Yes	Yes	Yes	Yes	Yes	Yes	Yes
Ross (2018), Australia	Yes	Yes	Yes	Yes	Yes	Yes	Yes

Supplementary Table 5. Quality and risk of bias assessment of quantitative and qualitative studies

**Author (Year), Location**	***MMAT criteria***											
	**S1**	**S2**	**QL1.1**	**QL1.2**	**QL1.3**	**QL1.4**	**QL1.5**	**QD4.1**	**QD4.2**	**QD4.3**	**QD4.4**	**QD4.5**

Bartik (2020), Australia	Yes	Yes	Yes	Yes	Yes	Yes	Yes	No	No	Yes	Yes	No
	**MM5.1**	**MM5.2**	**MM5.3**	**MM5.4**	**MM5.5**							
	Yes	Yes	Can't tell	No	No							
Spillane (2017), Ireland	**S1**	**S2**	**QL1.1**	**QL1.2**	**QL1.3**	**QL1.4**	**QL1.5**	**QD4.1**	**QD4.2**	**QD4.3**	**QD4.4**	**QD4.5**
	Yes	Yes	Yes	Yes	Yes	Yes	Yes	Yes	Yes	Yes	Yes	Yes
	**MM5.1**	**MM5.2**	**MM5.3**	**MM5.4**	**MM5.5**							
	Yes	Can't tell	Yes	No	Yes							

Supplementary Table 6. Quality and risk of bias assessment of mixed methods studies

**Category**	**Outcome**	**Relationship to deceased**	**Citation**	**Design**	**OR**	**CILL**	**CIUL**	**P value**	**Sample size (total)**
**Mortality**	Suicide mortality	Spouse or partner	Agerbo, 2005	Case-control	46.2 [a]	18.34	116.4	Not reported	189,231
			Erlangsen et al., 2017	Cohort	6.4 [b]	5.3	7.8	Not reported	7,006,898
			Jang et al., 2022	Cohort	3.871 [c]	3.165	4.733	<0.05	844,309 (families)
		Parent	Agerbo, 2005	Case-control	2.06 [a]	0.84	5.07	Not reported	189,231
			Jang et al., 2022	Cohort	1.817 [c]	1.464	2.256	<0.05	844,309 (families)
		Sibling	Rostila et al., 2014	Cohort	2.38 [d]	1.81	3.14	Not reported	1,743,088
		Any first-degree relative	Pitman et al., 2022	Case-control	3.26 [e]	2.62	4.06	Not reported	147,154

	All-cause	Sibling	Rostila et al., 2014	Cohort	1.26 [d]	1.14	1.4	Not reported	1,743,088
**Mental health and substance use**	Any mental health problem	Spouse or partner	Erlangsen et al., 2017	Cohort	1.8 [b]	1.6	2	Not reported	7,006,898
	Mood disorders	Spouse or partner	Erlangsen et al., 2017	Cohort	2.2 [b]	1.8	2.7	Not reported	7,006,898
	PTSD	Spouse or partner	Erlangsen et al., 2017	Cohort	12.1 [b]	6.4	22.7	Not reported	7,006,898
	Anxiety disorders	Spouse or partner	Erlangsen et al., 2017	Cohort	2.5 [b]	1.8	3.4	Not reported	7,006,898
	Deliberate self-harm	Spouse or partner	Erlangsen et al., 2017	Cohort	2 [b]	1.5	2.7	Not reported	7,006,898
	Alcohol use disorder	Spouse or partner	Erlangsen et al., 2017	Cohort	1.5 [b]	1.2	1.8	Not reported	7,006,898
	Drug use	Spouse or partner	Erlangsen et al., 2017	Cohort	1.7 [b]	1.2	2.5	Not reported	7,006,898
	Psychiatric hospitalisation	Spouse or partner	Erlangsen et al., 2017	Cohort	2.7 [b]	2.1	3.7	Not reported	7,006,898
	Initial psychiatric hospitalisation (no prior psychiatric conditions)	Family member	Cho et al., 2016	Cohort	2.665 [f]	1.495	4.75	Not reported	13,720
	Initial psychiatric hospitalisation (with prior psychiatric conditions)	Family member	Cho et al., 2016	Cohort	0.385 [f]	0.259	0.573	Not reported	13,720
	Recurrent psychiatric hospitalisation (no prior psychiatric conditions)	Family member	Cho et al., 2016	Cohort	2.135 [f]	1.425	3.198	Not reported	13,720

	Recurrent psychiatric hospitalisation (with prior psychiatric conditions)	Family member	Cho et al., 2016	Cohort	0.611 [f]	0.485	0.77	Not reported	13,720

OR: odds ratio; CILL; confidence interval lower limit; CIUL; confidence interval upper limit; PTSD; posttraumatic stress disorder.

[a] Adjusted for age, psychiatric admission, number of children, labour market affiliation, and education.

[b] Adjusted for age, year, socioeconomic status, marital status, number of children, number of siblings, and region.

[c] Adjusted for age.

[d] Adjusted for calendar period, country of birth age, civil status, income, existing physical conditions, prior psychiatric hospitalisation, and record of self-harm.

[e] Adjusted for marital status, family size, household income level, pre-bereavement history of self-harm, mental and physical health conditions.

[f] Adjusted for age, region, and socioeconomic status.

Supplementary Table 7. Summary of the effect estimates of psychosocial outcomes associated with suicide bereavement for men.
